# Supplementary figures and images for: Down-regulation of a cytokine secreted from peripheral fat bodies improves visual attention while reducing sleep in Drosophila
Source: PLoS Biol. 2020 Aug 3;18(8):e3000548. doi: 10.1371/journal.pbio.3000548 (PMC7426065; doi:10.1371/journal.pbio.3000548)

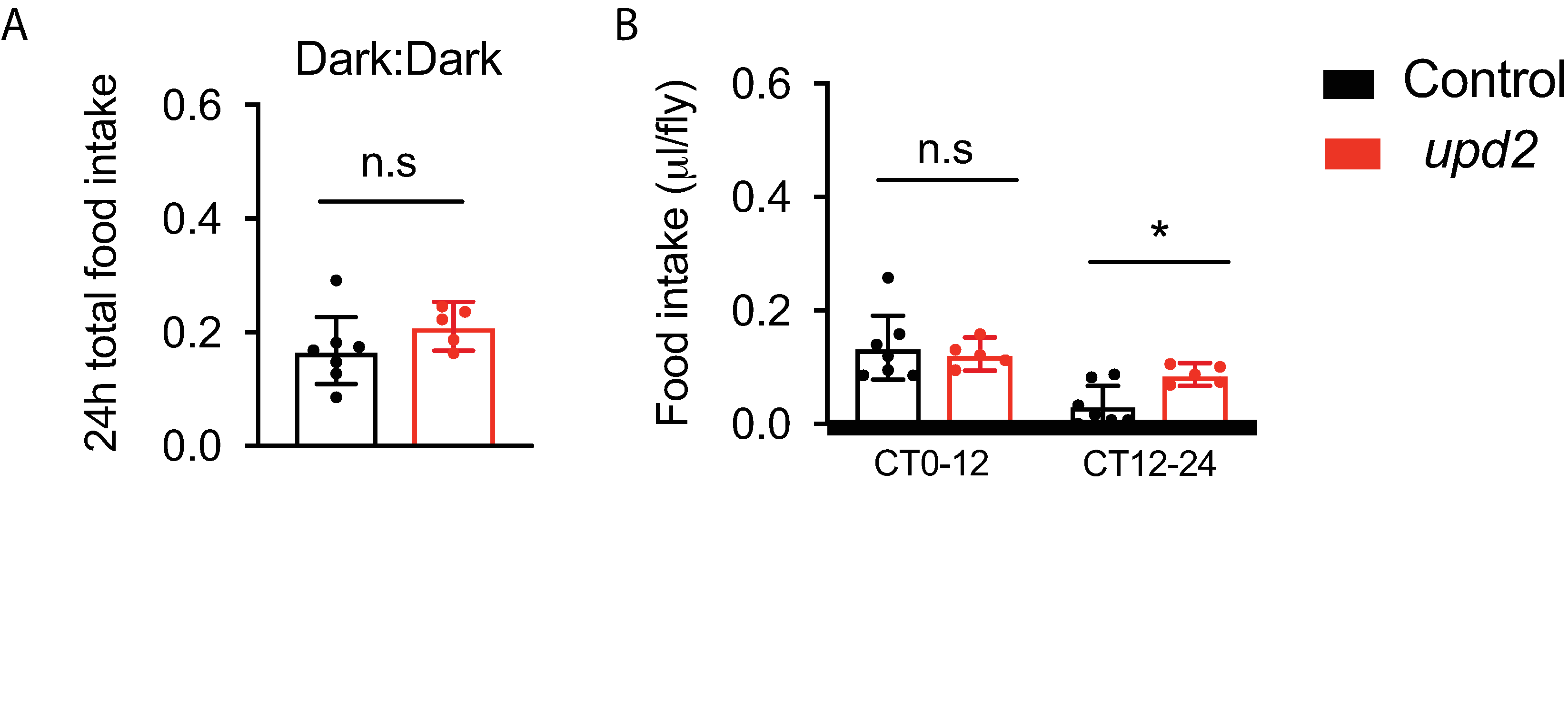

Supplement: S1 Fig — (A) Total food intake for feeding experiments under constant darkness (DD) was not significantly different between control and upd2 mutants. (B) upd2 mutants (red) had decreased nighttime food intake compared to controls (black); daytime food intake was similar to controls (n = 25–35 flies with 5 flies per Café chamber). *P < 0.05, **P < 0.01, ***P < 0.001, Student t test, error bars show SEM. The data underlying this figure can be found in S1 Data. Café, capillary feeding; upd2, unpaired 2. (TIF) [file pbio.3000548.s001.tif]

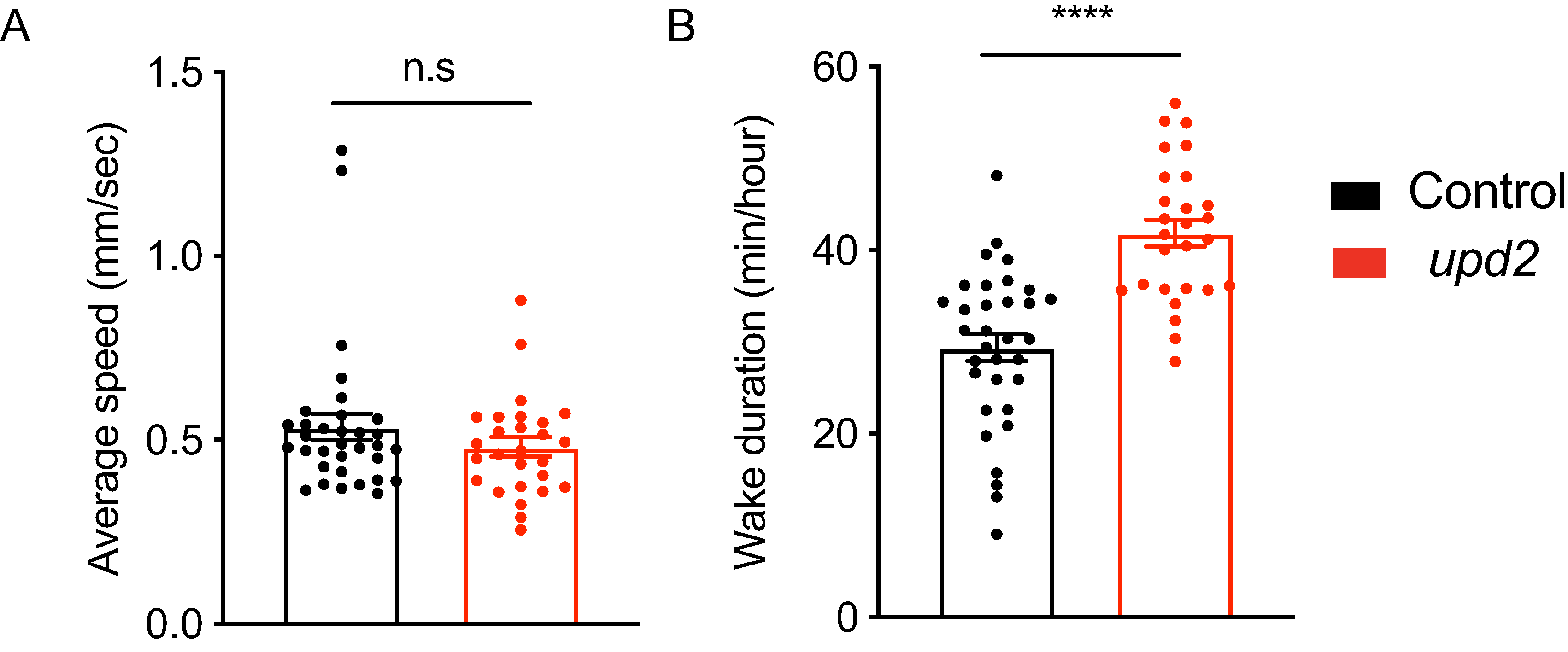

Supplement: S2 Fig — (A) Average speed of upd2 mutant flies (red) was not significantly different from controls (black). (B) Mutant flies had increased wake duration compared to controls. *P < 0.05, **P < 0.01, ***P < 0.001; flies in this figure are from the same data set as in Fig 1. Student t test for normally distributed data or Mann-Whitney U rank-sum test for nonparametric data was used to compare data sets. *P < 0.05, **P < 0.01, ***P < 0.001; error bars show SEM. The data underlying this figure can be found in S1 Data. upd2, unpaired 2. (TIF) [file pbio.3000548.s002.tif]

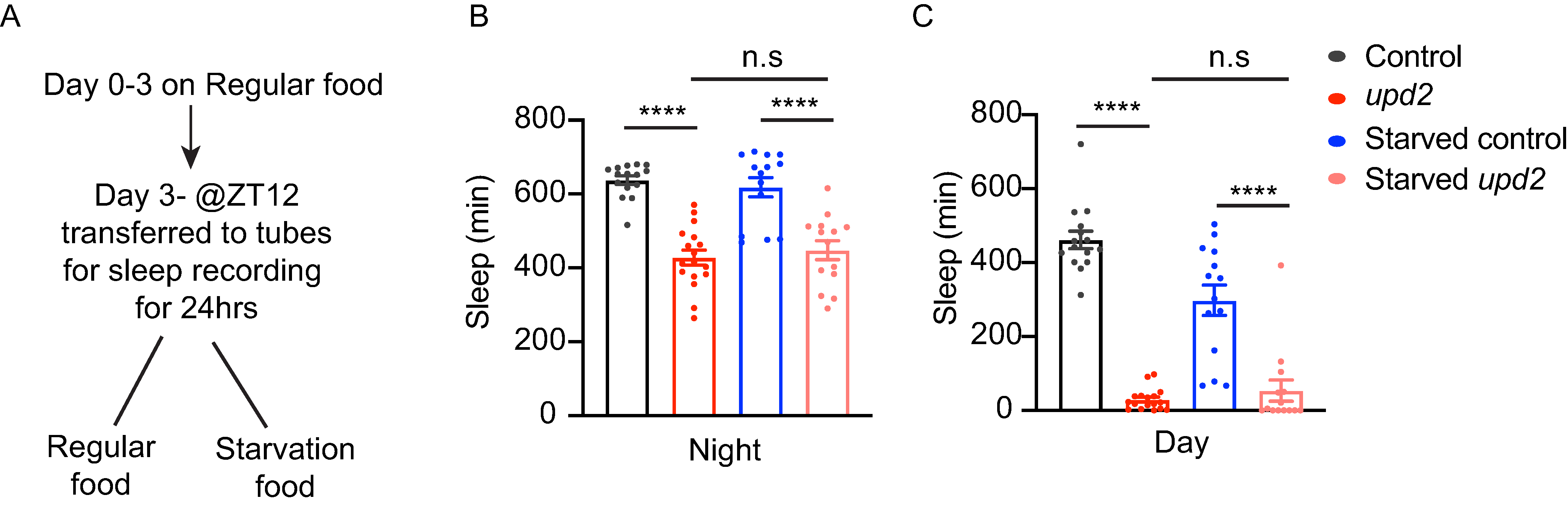

Supplement: S3 Fig — (A) Flies were kept on regular food from days 0–3. On day 3 at ZT12, they were placed into tubes with either regular food or starvation media for sleep tracking. Recording was started at nighttime and followed for 24 hours. (B) Both fed (red) and starved (pink) upd2 mutants slept significantly less during both night and (C) day compared to fed (black) and starved (blue) controls. n = 14–17, Student t test, *P < 0.05, **P < 0.01, ***P < 0.001, ****P < 0.0001; error bars show SEM. The data underlying this figure can be found in S1 Data. upd2, unpaired 2. (TIF) [file pbio.3000548.s003.tif]

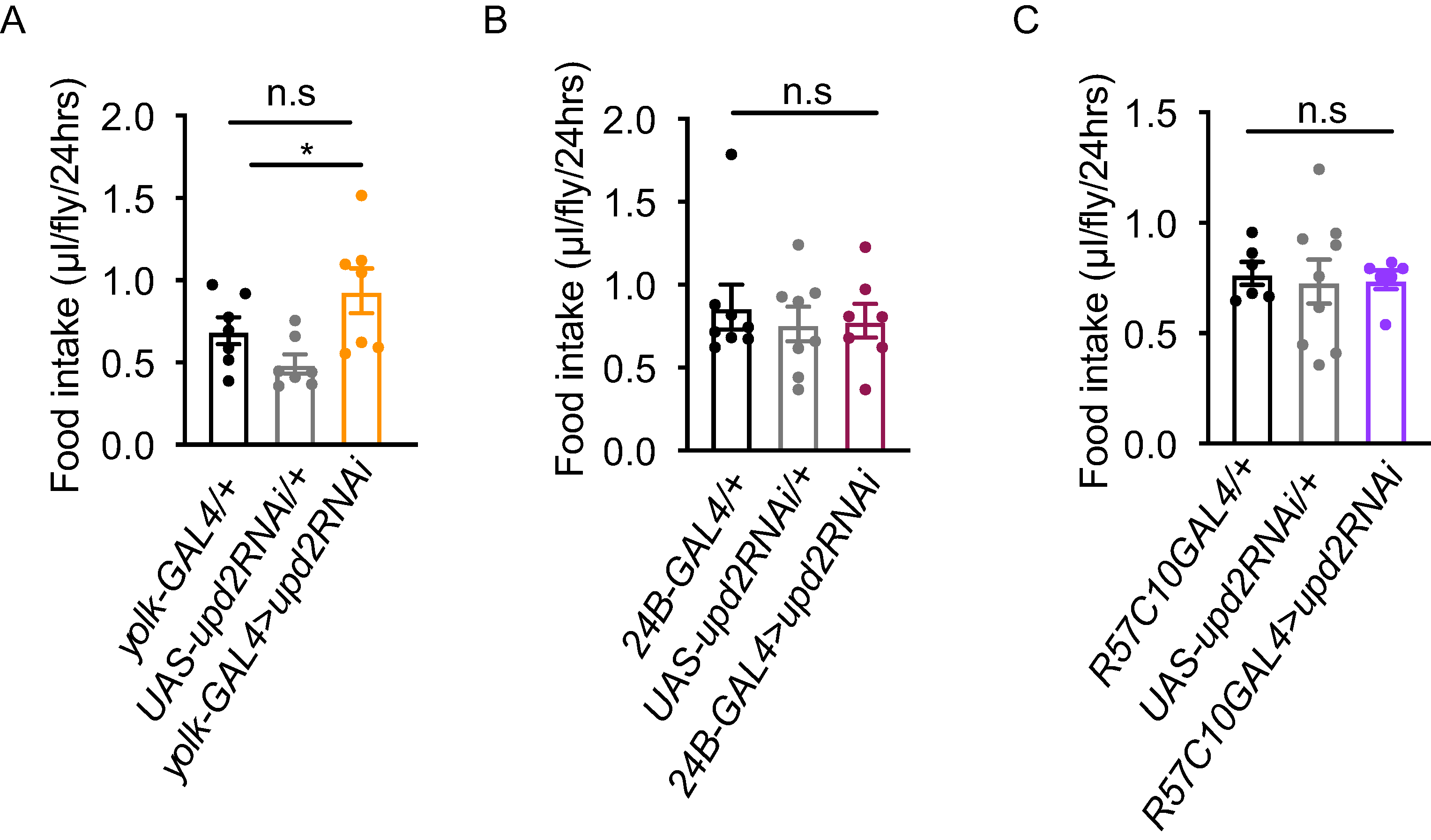

Supplement: S4 Fig — (A) FB knockdown of upd2 significantly increases food intake compared with UAS-upd2RNAi/+. (B,C) Muscle and pan-neuronal knockdown of upd2 shows similar food intake compared with both genetic controls. These data sets are the same as in Fig 2A, 2D and 2G. One-way ANOVA with Tukey correction was used for comparing different conditions. *P < 0.05, **P < 0.01, ***P < 0.001, ****P < 0.0001; error bars show SEM. The data underlying this figure can be found in S1 Data. FB, fat body; RNAi, RNA interference; UAS, upstream activation sequence; upd2, unpaired 2. (TIF) [file pbio.3000548.s004.tif]

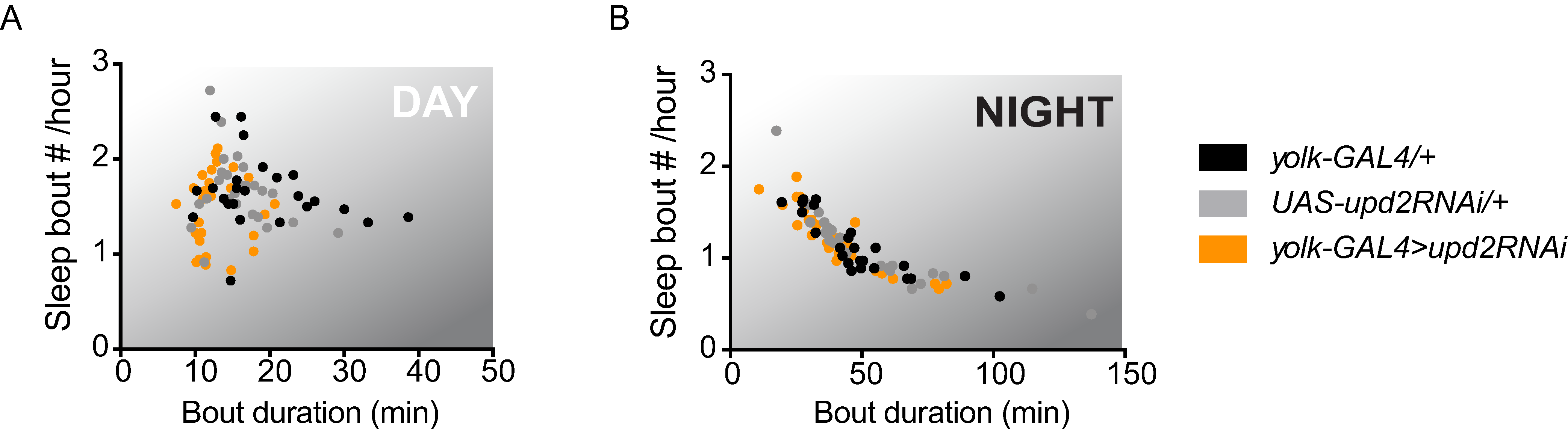

Supplement: S5 Fig — (A) Bout number plotted against average bout duration (minutes) showed that upd2 knockdown flies had fragmented day sleep. (B) Nighttime pattern was similar to controls (black, yolk-GAL4/+; gray, UAS-upd2RNAi/+; orange, yolk-GAL4>upd2RNAi). n = 24–28 per genotype; data set plotted here is the same as in Fig 2B and 2C. The data underlying this figure can be found in S1 Data. FB, fat body; GAL4, galactose-responsive transcription factor; RNAi, RNA interference; UAS, upstream activation sequence upd2, unpaired 2. (TIF) [file pbio.3000548.s005.tif]

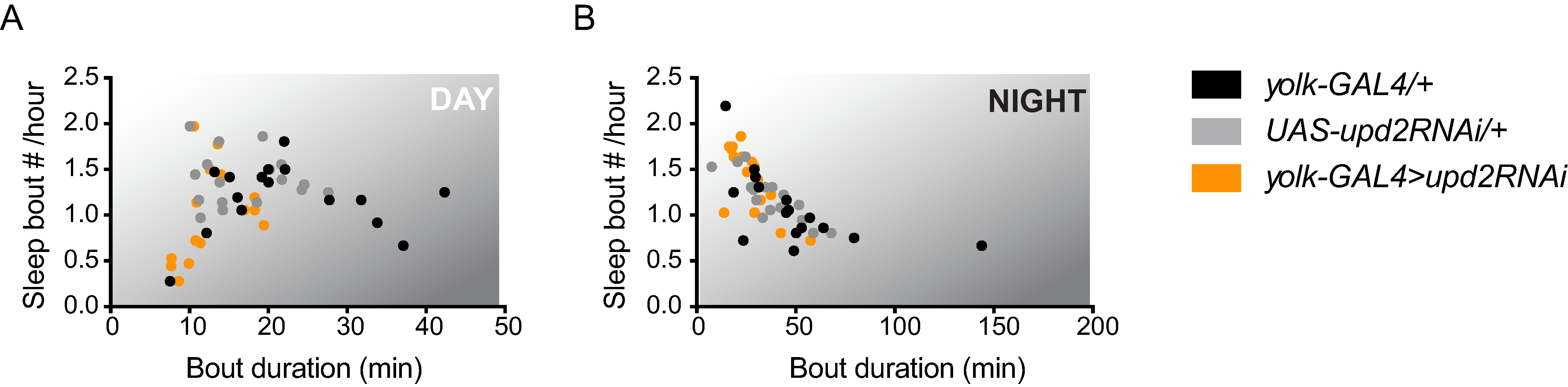

Supplement: S6 Fig — (A) Bout number plotted against average bout duration (minutes) showed a fragmentation pattern for daytime in open-field arena for upd2 knockdown flies. (B) Nighttime pattern was similar to controls. Sleep was tracked for 3 days (n = 15–17 per genotype) (black, yolk-GAL4/+; gray, UAS-upd2RNAi/+; orange, yolk-GAL4>upd2RNAi). Data set plotted here is the same as in Fig 4. The data underlying this figure can be found in S1 Data. FB, fat body; GAL4, galactose-responsive transcription factor; RNAi, RNA interference; UAS, upstream activation sequence; upd2, unpaired 2. (TIF) [file pbio.3000548.s006.tif]

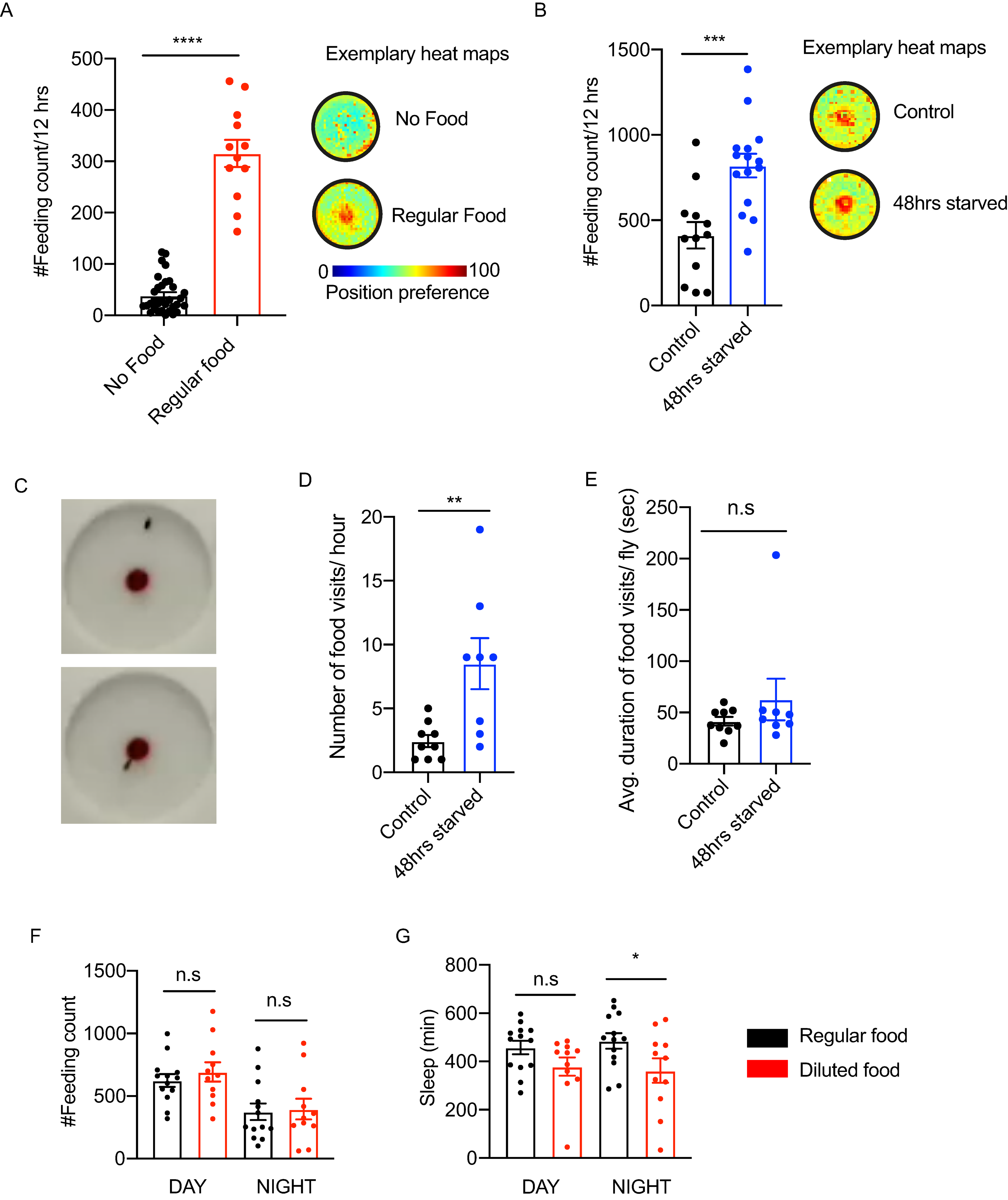

Supplement: S7 Fig — (A) Number of feeding events with access to the food cup (red) compared with “no food” condition (black), where the food cup was covered with parafilm to prevent access. Exemplary heatmaps for flies under different food conditions (right panel). (B) Flies starved for 48 hours (blue bar) displayed a significant increase in feeding counts compared with control flies that had been fed (black) (n = 13–15). (C) Representative images from the video recordings. The bottom image is showing a fly feeding. (D) Visual annotation of the number of food visits displayed a significant increase in starved flies. (E) The average duration of food visit per fly was not significantly different between control and starved flies. (F) Flies on diluted food (red) (20% of regular food calories) displayed no change in their feeding counts compared with flies on regular food (black) but (G) slept less during both day and night (n = 10–13, Student t test for normally distributed data or Mann-Whitney U rank-sum test for nonparametric data was used to compare data sets. *P < 0.05, **P < 0.01, ***P < 0.001; error bars show SEM. The data underlying this figure can be found in S1 Data. (TIF) [file pbio.3000548.s007.tif]

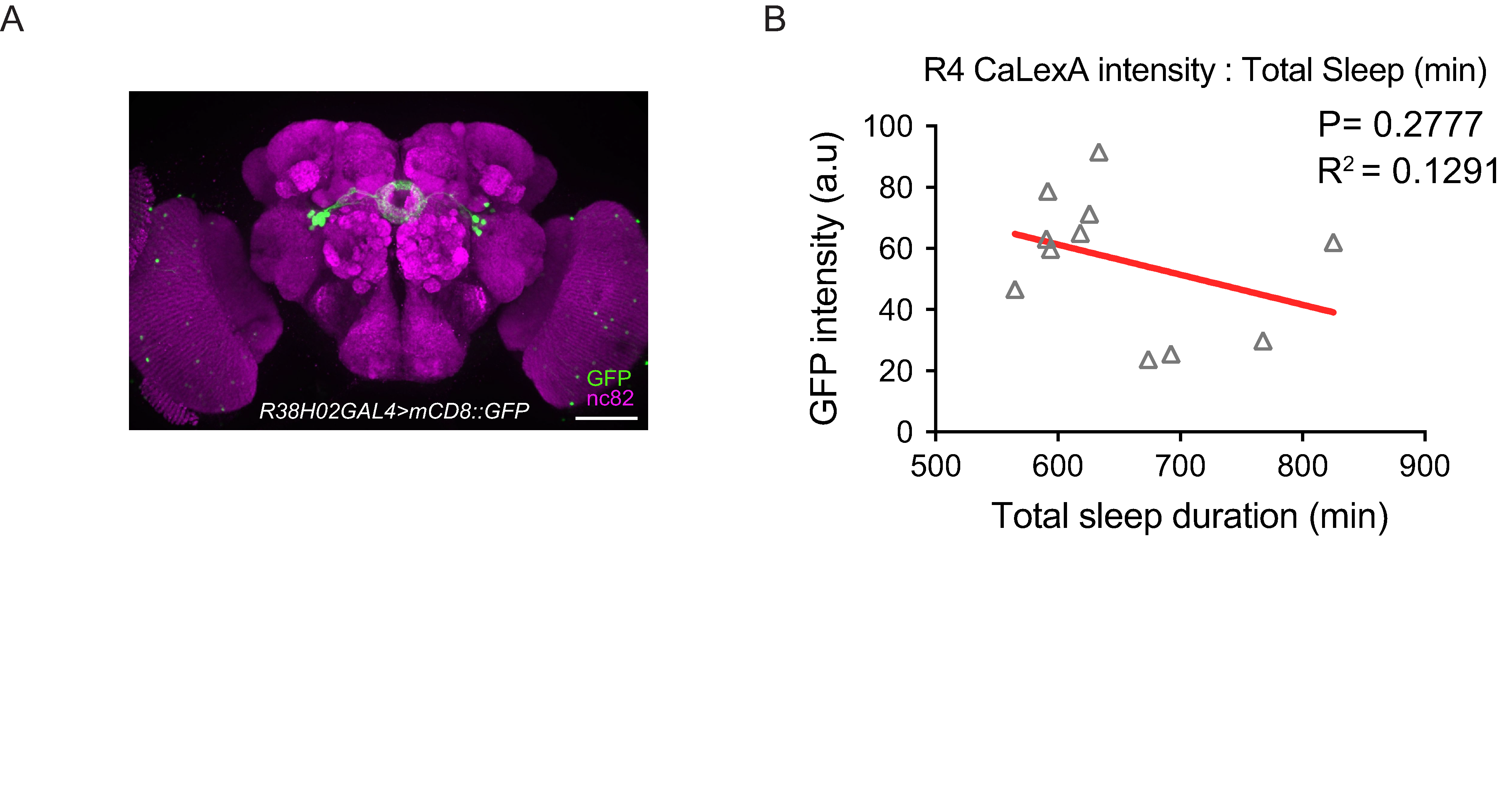

Supplement: S8 Fig — (A) Expression of R38H02-GAL4 in the brain using UAS-mCD8::GFP (green). Neuropil is stained with bruchpilot (nc82, magenta). Scale bar, 100 μm. (B) CaLexA intensity of individual flies plotted against their total sleep duration (over 24 hours). Flies were housed in open-field arenas. Two-tailed P values for Pearson’s correlation coefficient are shown. Analyses in this figure is from the same data set as in Fig 5. The data underlying this figure can be found in S1 Data. CaLexA, calcium-dependent nuclear import of LexA; GAL4, galactose-responsive transcription factor; GFP, green fluorescent protein; UAS, upstream activation sequence (TIF) [file pbio.3000548.s008.tif]

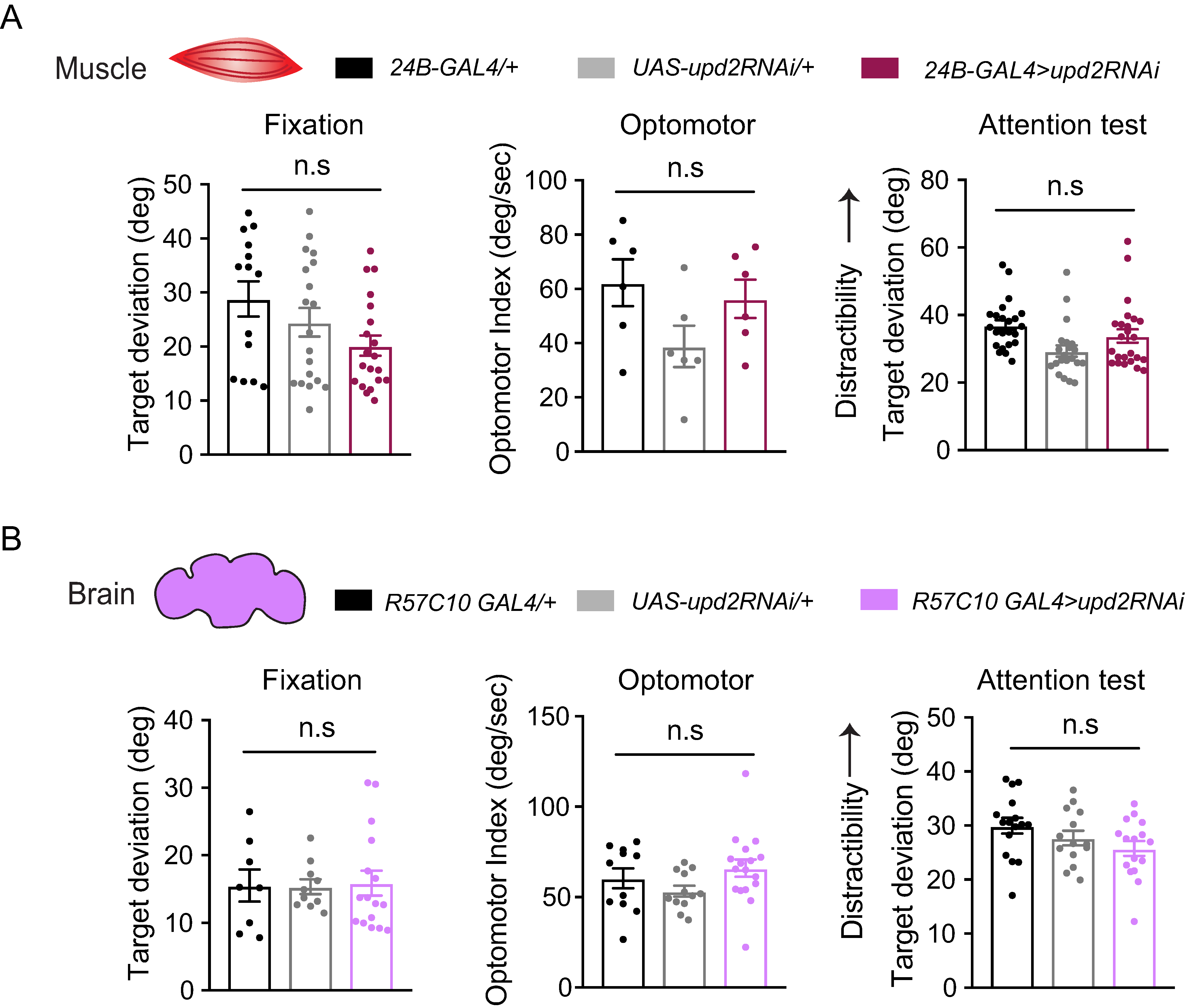

Supplement: S9 Fig — (A) We did not observe any differences in simple visual behaviors (fixation [n = 15–20], optomotor [n = 6–8] or in visual attention [n = 13–16] with muscle-specific upd2 knockdown (24B-GAL4>upd2RNAi, maroon) compared with controls (24B-GAL4/+, black and UAS-upd2RNAi/+, gray). (B) Pan-neuronal knockdown of upd2 (R57C10-GAL4>upd2RNAi, purple) also had no impact on visual behaviors (optomotor, n = 12–16), fixation, n = 8–16), and visual attention (n = 14–16) compared with genetic controls (R57C10-GAL4/+, black and UAS-upd2RNAi, gray). One-way ANOVA with Tukey correction was used for comparing different conditions. *P < 0.05, **P < 0.01, ***P < 0.001, ****P < 0.0001; error bars show SEM. The data underlying this figure can be found in S1 Data. GAL4, galactose-responsive transcription factor; RNAi, RNA interference; UAS, upstream activation sequence; upd2, unpaired 2. (TIF) [file pbio.3000548.s009.tif]

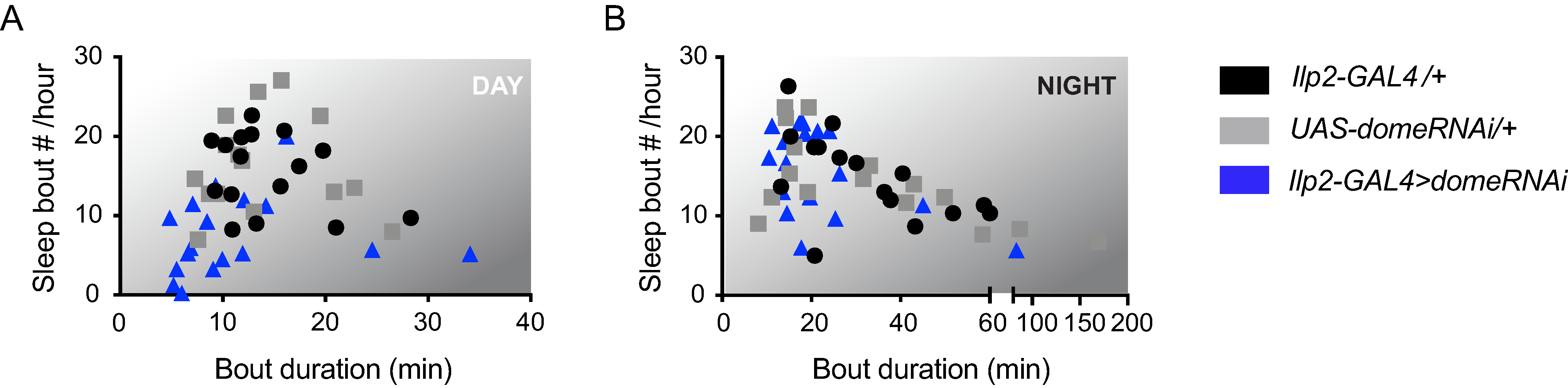

Supplement: S10 Fig — (A) Bout number plotted against average bout duration (minutes) showed a fragmentation pattern for daytime. (B) There was no obvious fragmentation pattern for nighttime. Flies in this figure are from the same data set as in Fig 8D and 8E. Sleep was tracked for 3 days (n = 15–17 per genotype). (Ilp2-GAL4/+, black; UAS-domeRNAi/+, light gray; blue Ilp2-GAL4>domeRNAi. n = 15–17 per genotype. Sleep was recorded over 3 days. The data underlying this figure can be found in S1 Data. dome, domeless; GAL4, galactose-responsive transcription factor; Ilp2, insulin-like peptide 2; RNAi, RNA interference; UAS, upstream activation sequence. (TIF) [file pbio.3000548.s010.tif]

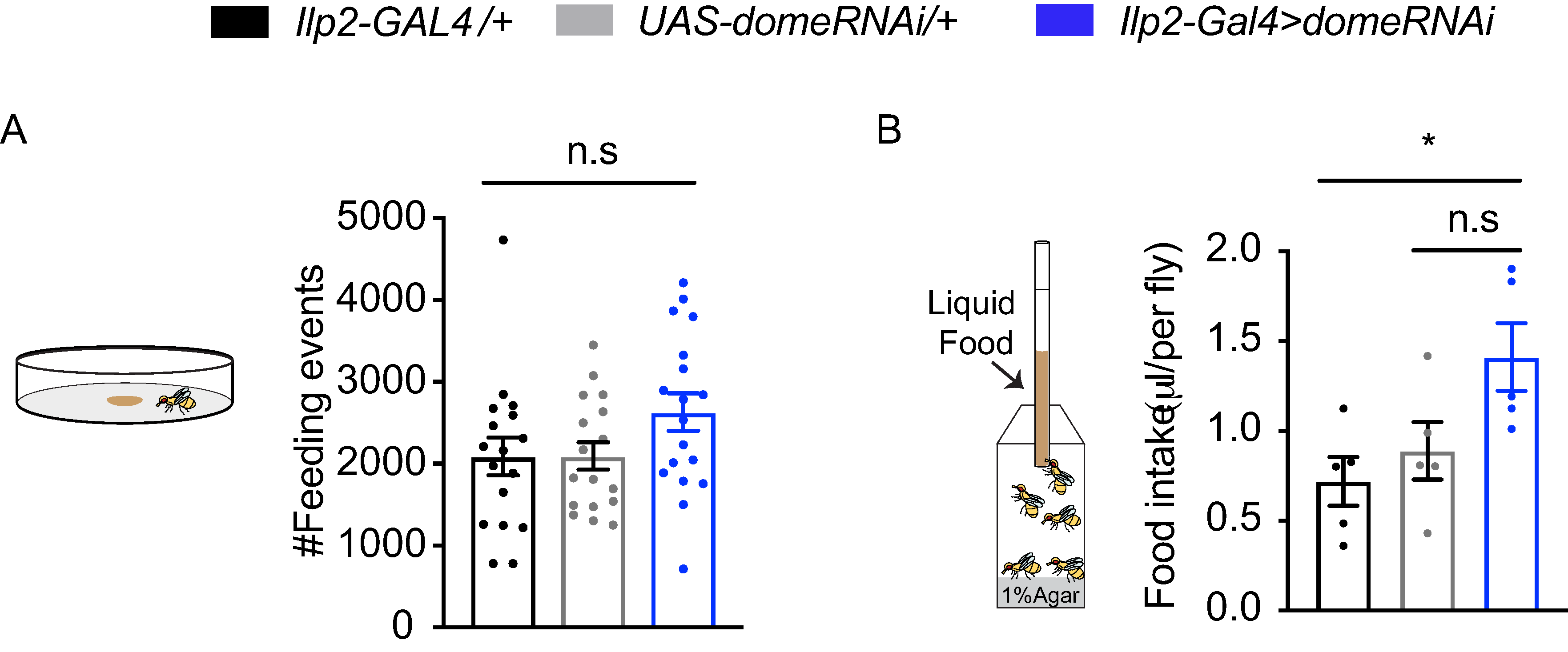

Supplement: S11 Fig — (A) Number of feeding events was not significantly different between control flies and dome knockdown flies. Flies in this figure are from the same data set as in Fig 8D and 8E. (B) Total food intake over 24 hours in Café chamber of dome knockdown flies was significantly increased compared to one of the genetic controls (n = 25, with 5 flies per chamber). One-way ANOVA with Tukey correction was used for comparing different conditions. *P < 0.05, **P < 0.01, ***P < 0.001, ****P < 0.0001; error bars show SEM. The data underlying this figure can be found in S1 Data. Café, capillary feeding; dome, domeless. (TIF) [file pbio.3000548.s011.tif]

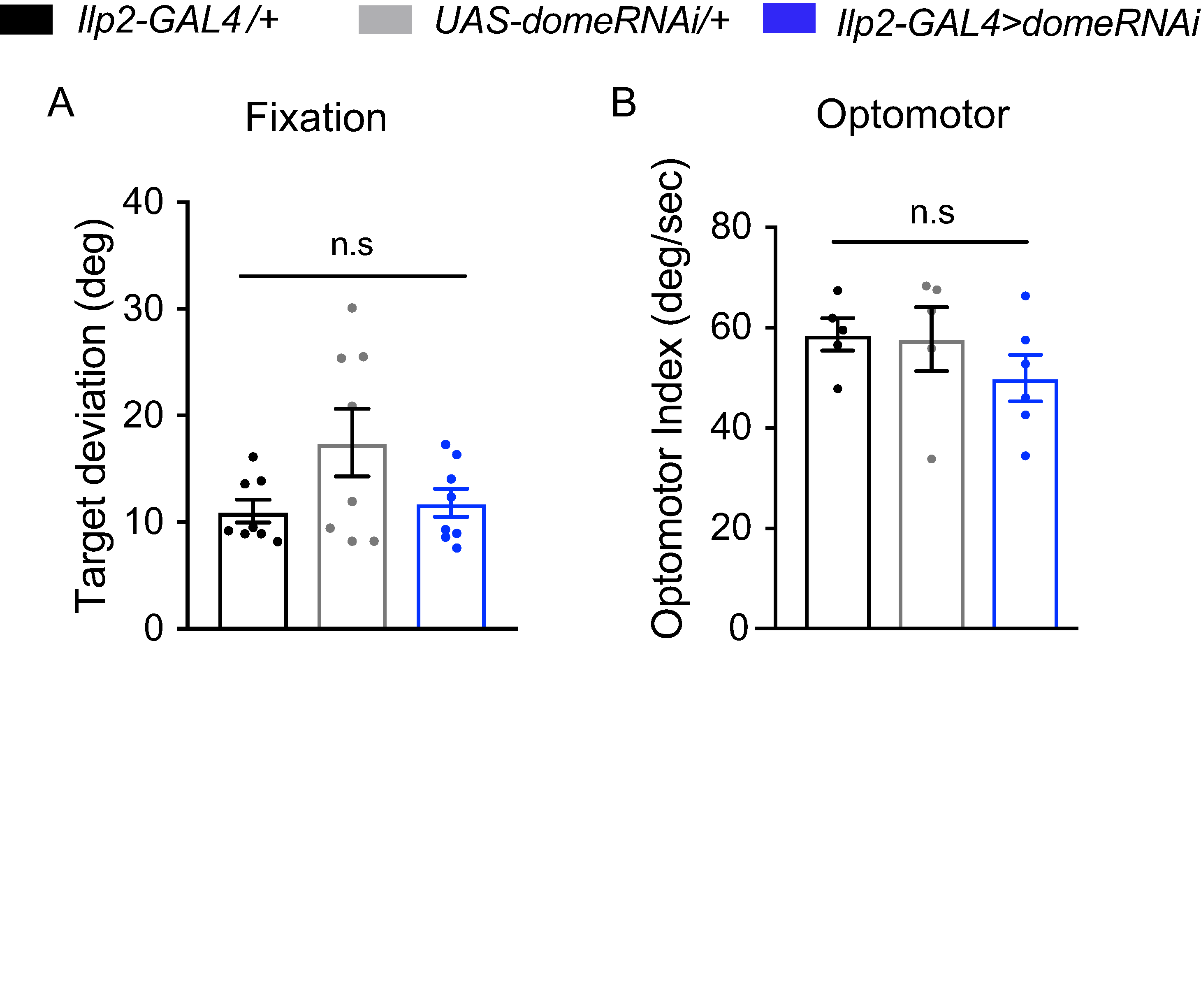

Supplement: S12 Fig — (A) Fixation and (B) optomotor behavior of Ilp2-GAL4>domeRNAi (blue) were not significantly different from Ilp2-GAL4/+, black, and UAS-domeRNAi/+, gray. n = 6–8 per experiment; one-way ANOVA with Tukey correction was used for comparing different conditions. *P < 0.05, **P < 0.01, ***P < 0.001, ****P < 0.0001; error bars show SEM. The data underlying this figure can be found in S1 Data. dome, domeless; GAL4, galactose-responsive transcription factor; Ilp2, insulin-like peptide 2; RNAi, RNA interference; UAS, upstream activation sequence. (TIF) [file pbio.3000548.s012.tif]
