# Supplementary material for: Down-regulation of a cytokine secreted from peripheral fat bodies improves visual attention while reducing sleep in Drosophila
Source: PLoS Biol. 2020 Aug 3;18(8):e3000548. doi: 10.1371/journal.pbio.3000548 (PMC7426065; doi:10.1371/journal.pbio.3000548)
Supplement: Attachment — Submitted filename: Reply to Reviewers B.docx [file pbio.3000548.s014.docx]

We thank the Reviewers for their helpful comments. As there was some overlap between what the different Reviewers requested, we addressed these more thoroughly upon first mention in our reply (in blue text) below. We have attempted to address all of the Reviewers’ concerns, either in new experiments, in re-analyzed data, or in the manuscript text.

**Reviewer # 1**

Ertekin et al report that fat body-specific knockdown of upd2, which was previously shown to be downregulated during starvation and increased with high-fat and high-sugar diets, increases feeding, decreases sleep, and enhances visual attention. Using a new tool for simultaneously assessing sleep and feeding-related behavior in individual flies in combination with a neuronal activity reporter, the authors show that activity of R4 neurons of the ellipsoid body are elevated in food-deprived and in upd2 mutants. Considering the prominent effect of sleep on cognitive function and the widespread practice of starving flies prior to behavioral tests, their finding that a manipulation that mimics starvation and suppresses sleep can improve performance on a cognitive task is novel, exciting, and may profoundly impact the way researchers design experiments and interpret their data. However, some of the conclusions in this manuscript are based on untested assumptions or only indirectly supported by the data. I believe that additional critical experiments and thoughtful rephrasing can greatly improve the cohesiveness and logical flow of this manuscript.

Major comments:
1) Some of the text used throughout the manuscript should be revised to more accurately reflect the findings. a) “Sleep need”: Data presented show that upd2 downregulation decreases sleep, but whether it also decreases sleep need per se has not been tested or shown. The authors should be conservative when referring to the reduced sleep phenotype of upd2 mutants and KDs. Alternatively, the authors could add additional experiments to support the idea, e.g., a thermogenetic approach could be used to show that flies do not sleep rebound after the downregulation has been lifted. b) Synonymously referring to upd2 as a “satiety signal” or downregulation of upd2 as “starved-like state”: Most of the manuscript does not directly compare the effect of upd2 manipulation with that of food deprivation (except in one instance, for R4 neuronal activity). I suggest avoid generalizing upd2 downregulation as “a lack of satiety cue” or “starvation-like state,” except in the Discussion.

The Reviewer is correct in stating that our chronic manipulations do not necessarily suggest increased sleep need, only that upd2 downregulation decreases sleep duration and intensity. We have therefore adjusted our language (starting with the title) to reflect this and simply refer to ‘upd2 downregulation’, up to the R4 CaLexA experiments. At that point, we observe a correspondence between increased R4 activity in starved flies and in an *upd2* mutant background. We feel that is therefore reasonable to allude to a starved-like state thereafter in the results, and then reserve speculations about a satiety cue to the discussion as proposed. Regarding thermogenetic experiments, the fat body driver (Yolk-Gal4) only expresses in adults (Georgel, 2001; Barrio et al, 2014) so ruling out developmental effects is not necessary. We also hesitate to do any thermogenetics in the adults because of known effects of extended heat exposure on sleep. Our *upd2* knockdown results are supported by the *domeless* receptor knockdown experiments, which confirm that *upd2* signalling to the brain regulates these sleep, feeding, and visual attention phenotypes. We feel that this is a sufficient finding for our study but concede that our language was perhaps too specific.

2) Since the fat body and upd2 are central to the main findings of the manuscript, the driver and the RNAi should be validated. Fat body drivers are notoriously non-specific, and many publications use two or more fat body drivers to confirm a fat body-specific effect. Alternatively, or in addition, the authors could cite or show expression data for the driver. Regarding the RNAi, it’s common practice to use two independent RNAi constructs, and there are several other RNAi lines with no predicted off-target effects that are available for upd2, including another TRiP short hairpin. Finally, to more rigorously support the genetic interpretations, the authors could test whether fat body-specific expression of upd2 can rescue the feeding and sleep phenotypes in upd2 mutants.

It is true that fat body drivers can be non-specific. However, the one we employed (Yolk-Gal4) is precise and expression data has been published (Georgel et al., 2001), which we reference. Others have shown that Yolk-Gal4 expression begins in adulthood (Barrio et al, 2014), and we have confirmed this in our lab. We feel that it is not necessary to show confirming figures of this in our paper as this is not novel, but will do so if required.

As proposed by the Reviewer, we have now tested a second TRiP short hairpin RNAi line #33949 (BDSC) and observed a similar but less strong phenotype for both sleep and attention. That this is a weaker RNAi construct has been observed previously (Yurgel et al., 2018). Importantly, the phenotypes are both in the same direction as for our first RNAi construct (#33988). We show the data for the second RNAi below, in **Figure R1**. We prefer to keep these data in the Reply letter, as this should be made available to eventual readers.

As suggested by the Reviewer, we prepared crosses to test for rescue of the upd2 mutant, by using a UAS-*upd2* which we acquired from J. Hombria (Ref. #33). Unfortunately rescue experiments were genetically problematic as the *upd2* overexpression construct led to lethality in adults when it was combined with Yolk-Gal4. We now mention this in the text (lines 209-210). We feel that this setback does not compromise our conclusions, because our *upd2* mutant results are complemented by specific upd2 knockdown experiments in Yolk-Gal4, and *domeless* knockdown in ilp2 cells, with consistent phenotypic data throughout.

**Figure R1: Sleep and attention phenotype of upd2 knockdown, using a second RNAi line (#33949)** Sleep was tracked for 3 days. Yolk-Gal4/+, black and UAS upd2 RNAi/+, grey;Yolk Gal4> UAS upd2 RNA, orange (n=11-12). One-way Anova with Tukey correction was used for comparing different conditions. *P <0.05, Error bars show SEM.

3) Regarding the feeding estimates from the open-field platform, I think the authors are actually measuring a food-dwelling or food-proximity behavior, and the authors should be careful about calling it “feeding” or “feeding activity.” It certainly isn’t “food intake.” Other available methods of feeding estimation (e.g., FLIC, flyPAD) have undergone extensive efforts to validate that the estimates correlate with actual food consumption. Figure S3 is not evidence for the accuracy of feeding estimates, since it only shows that the measurements can distinguish behaviors in the presence and absence of food. Whether the assay can resolve different levels of food intake or feeding-related behaviors—and thus can be used for quantitative comparisons—needs to be tested. Some examples of additional experiments that may be useful include: a) Do the feeding estimates increase with prolonged starvation; b) Can the assay resolve a compensatory feeding response to food dilution; and c)
Correlate their estimation with an actual feeding measurement (e.g., see the flyPAD paper, Figure 3 of Itskov et al. (2014) Nat. Comm.)

We thank the Reviewer for their suggestions on how to better validate feeding in our open-field platform. In our original submission, we were confident that we were measuring actual feeding (quantified as feeding counts) for a number of reasons, which included visual inspection as well as covering or modifying the food. We also showed a significant negative correlation between R4 activity (which has been found to be an indicator of feeding status) and feeding counts among individual flies (Fig. 5B). Such a correlation would not have emerged if flies were simply dwelling near the food without eating it; there was no correlation with sleep, for example (Fig. S8B). Finally, the feeding behaviour in *upd2* knockdown animals mirror closely food consumption for the same strain in the Café assay (compare Figure 4F with 2A). Nevertheless, to further convince ourselves that our feeding count estimates were relevant, we assayed starved flies as proposed, and feeding counts were significantly increased in this case (these data are now in **Fig. S7B**). We also performed the food dilution experiment as proposed and found no difference in average feeding counts. However, when we examined sleep duration, we observed that flies eating diluted food slept less (these data are now in **Fig. S7C,D**). This is consistent with the ‘diluted’ flies being semi-starved. Together, our new results suggest a ceiling effect in how much flies eat in our open-field paradigm (hence no difference in feeding counts between the diluted and undiluted group), but flies do eat more when they have been starved for 48 hours (hence a significant difference in our starved data). It should also be noted that there is considerable individual variability, which is evident in all our data. Our open field paradigm captures this individual variability, which is how we could relate individual feeding counts to R4 neuronal activity. Together, we feel that all of these experiments and observations convincingly show that our analysis of fly behaviour near the food cup (it isn’t just dwell time, as outlined in the Methods and now also in the text, lines 322-326) accurately estimate feeding behaviour. We however now make sure to call this ‘feeding-related behaviour’ throughout the manuscript. This is an accurate description, and a relevant readout for the purposes of our behavioural study: *upd2* knockdown animals show *more* feeding-related behaviour.

4) Figures 6 and S6: THIP’s sleep-inducing effect is validated in upd2 mutants but its effect on visual attention is tested in fat body upd2 knockdowns. The pharmacological validation and the phenotype measurements should be performed with the same genetic manipulation. Did upd2 KD flies also sleep more with THIP? Alternatively, did upd2 mutants also perform better than the controls in the visual attention task, with and without THIP? If the authors wish to support the idea that it is the starved state that enhances performance in the visual attention test, they need to show that food deprivation also improves visual attention in genetic controls. Without this validation, it is difficult to determine whether it is the “starvation-like state” or something entirely different that enhances visual attention of the upd2 KD flies.

We repeated our THIP experiments on the *upd2* knockdown and its genetic controls, as requested. These new sleep and attention data are now shown as a main figure (**Fig. 7**) to better match the preceding attention experiments in Fig. 6, which (as correctly pointed out by the reviewer) are knockdown experiments rather than mutants. It is evident from our new data that THIP exposure significantly increases sleep in genetic controls as well as *upd2* knockdown (KD) animals, as expected. Interestingly, although the increase in sleep is dramatic for all, sleep in KD animals is not increased to exactly the same level as in genetic controls. This suggests that the ‘genetically starved-like state’ in KD animals prevents achieving quite as much increased sleep as in the genetic controls. To check that a starvation signal might be relevant here, we did an additional experiment: we fed THIP to starved wild-type flies, to see if these also slept less than controls. This is indeed what we saw, confirming that THIP-induced sleep is somewhat blunted under a starvation regime (**Figure R2**, below). Since this is not immediately pertinent to our study, we have decided not to include Figure R2 in our revised manuscript. More importantly, all of our THIP data are now in the same strains, confirming that even with increased sleep, *upd2* KD flies still maintain their attention phenotype. This is a very important point, as it suggests that the attention phenotype is not only due to insufficient sleep in *upd2* KD animals.

**Figure R2. THIP administration on starvation medium decreases the efficacy of sleep induction**

(A) Sleep profile of flies over 24 hours. (B) Total sleepquantifications. Starvation medium was 1%Agar . THIP (0.1 mg/ml) was administered 12 hours prior and during sleep recordings. N=14-17 per group. One-way Anova with Tukey correction was used for comparing different conditions. *P <0.05, **P <0.01, ***P <0.001, ****P<0.0001 Error bars=SEM.

Regarding the second question about acute starvation ‘improving’ attention, we did the starvation experiment as proposed , in both genetic controls as well as the knockdown animals (see **Figure R3**). We were first surprised to find that acute starvation seemed to abolish the improved attention in *upd2* KD animals, compared to genetic controls (Figure R3A). However, locomotion (quantified by distance travelled) was reduced in KD animals (Figure R3B), suggesting that this effect is probably due to the general ill health of these flies following starvation. This resonates with our reasoning why we used ‘genetic starvation’ as a better approach, as other factors related to metabolism and poor health following acute starvation could interfere with our ability to properly dissect this problem. Thus, we suspect that after 24 hours of starvation, we are not really looking at a ‘cognitive’ phenotype (also in controls) but a nonlinear mixture of physiological effects over which we have little control. In contrast, the *upd2* KD animals are well fed, allowing us to study this pathway while sidestepping likely metabolic effects. Importantly, locomotion in *upd2* KD animals is normal, not different from genetic controls (see **Figure R4**), even though their attention is different. Thus, perhaps counterintuitively, acute starvation experiments probably confuse the issue, because flies become defective for other reasons. Exactly how much starvation is beyond the current scope of this study. We hope that this is a satisfying explanation. We now allude to this confounding issue in the discussion (lines 537-543).

**Figure R4. Visual attention (A) and locomotion (B) in well-fed upd2 knockdown flies and genetic controls**.

(These are flies from data presented in the manuscript in Figure 6D)

**Figure R3. Visual attention (A) and locomotion (B) in starved upd2 knockdown flies and genetic controls**. (Yolk-Gal4/+, black and UAS upd2 RNAi/+, grey;Yolk Gal4> UAS upd2 RNA, orange (n=8 per genotype) One-way Anova with Tukey correction was used for comparing different conditions. *P <0.05, n.s., not significant. Error bars show SEM.

5) R4 neurons as a neural correlate of starvation: This experiment doesn’t integrate well with the rest of the manuscript. The increased activity of R4 in the upd2 mutants is not connected to phenotypes other than starvation, including decreased sleep and enhanced visual attention. Also, because the relationship between starvation and increased R4 activity is not further studied, the experiment only raises more questions. I think excluding this section entirely would improve the cohesiveness of the manuscript.

The R4 CaLexA experiments are there to implicate a starved-like state in *upd2* mutants, consistent with published work (Park et al., 2016). This was never intended as an entry point into circuit analysis of R4 function in sleep, feeding, or attention. We would therefore prefer to keep these results in the paper, as they lend credence to our view that *upd2* mutants are acting on a genetically-induced starvation cue, even though they are well fed. This provides a crucial transition point for our paper, where can start considering the possibility of a starvation signal. We are of course aware that the R4 neurons aren’t just that. They probably shape visual behaviours and motivation more generally, which is another story.

Alternatively, some suggestions for making this finding more substantiated and relevant include:

a) Rule out a contribution from sleep suppression on R4 activity. Both starved flies and upd2 mutants sleep less than controls. Although the authors show that the R4 activity is not significantly correlated with the range of sleep durations in freely-sleeping, well-rested flies, an “abnormal” level of sleep may still affect R4 activity. The authors should measure R4 activity in sleep-deprived controls and compare it to fed or starved controls and the upd2 mutants. For a more thorough dissociation experiment, the authors could additionally compare sleep-deprived/starved controls with sleep-deprived/fed or rested/starved controls to test whether the effects of sleep- and food-deprivations on R4 activity are additive.

The Reviewer is proposing that the R4 neurons may be lighting up because of abnormally low sleep, rather than nutritional cues. While it remains possible that increased R4 activity reflects abnormally low sleep (thus not detected by our correlation), the link with starvation is accepted, from previously published work (Park et al, 2016). Further, it is questionable whether the *upd2* mutants are actually sleep deprived (we now discuss this, lines 577-599), so we question sleep deprivation experimens to address this. We agree however about the value of investigating possible additive effects, so we redid the CaLexA experiment on starved *upd2* mutants as suggested. These showed a similar (increased) level of R4 activity. These data have been added to the CaLexA figure (**Figure 5D,E**).

b) To support the idea that R4 activity level correlates with nutrition state, the neuronal activity should be correlated with actual food intake instead of extended time spent on food, especially since the “feeding estimates” in the open-field arena has not been validated.

We hope that our validation arguments and additional experiments, discussed above, are convincing now. Feeding estimates are not just based on dwell time near the food; there are other criteria involved. Also, if our software was not correctly estimating feeding events, then there would not have been any correlation with R4 activity. It is difficult to imagine another reason why such a (negative) correlation might have emerged, if not for what flies were probably doing while on the food, namely feeding. In this regard, our open field feeding/sleep paradigm is valuable, as it disambiguates more effectively than a linear paradigm (i.e., Trikinetics tubes) actual feeding events from dwell time near a food source. We have clarified now throughout our manuscript that we are estimating feeding-related behaviour.

c) It is unclear why the feeding parameter being correlated with R4 CaLexA intensity is % time spent feeding rather than the number of feeding events, when the latter is the metric the authors use everywhere else when analyzing feeding behavior in the open-field arena (Figures 4, 7, S3, S8). Does the number of feeding events also correlate with R4 activity?

We thank the Reviewer for detecting this oversight. We have corrected the metric in Figure 5B, so it is the same as elsewhere. All of our feeding metrics are now # of feeding events, which as stated above derives from a number of other metrics (actually 4, see Methods, lines 667-672), not just dwell-time near the food. This metric is available in the DART software.

d) The relationship between feeding and R4 activity can be tested easily and more definitively using optogenetic stimulation of R4 neurons, similarly to Figure S5 in which the authors show that optogenetic activation of R4 decreases sleep. If R4 activity reflects starved state, does artificially activating R4 neurons increase food intake?

As discussed above, we hesitate to make causality tests for the R4 neurons central to this study. Rather, R4 activity gave us confidence that we could consider this a starved-like state, nothing more. We are not trying to establish acute causality for the R4 neurons here, they just serve as a marker of a chronically starved-like state. To better promote this consistent narrative, we have now removed the optogenetic experiments (old Figure S5) from our manuscript. These experiments were out of step with our narrative. The fact that optogenetic activation of R4 neurons decreased sleep is not central to our story, and stood out as the single acute experiment in what is really a story about a chronic genetic condition. We do not want to mislead readers by suggesting that the R4 neurons are causal to the various phenotypes we have observed. They serve as a validation linked to published starvation work.

e) If the R4 neurons are THE neural correlates of starvation, and starvation is the cause of enhanced visual attention, then does R4 activation enhance visual attention in a similar way?

Please see our answer regarding acute activation of the R4 neurons, above. We nevertheless thought that this was an interesting question, because ellipsoid body neurons have been implicated in visual processing in *Drosophila*. Specifically, we wondered if activation of the R4 neurons might improve attention. Unfortunately, we haven’t had much success in applying optogenetic approaches to our attention arena for freely walking flies, perhaps because of light interference. Instead, we crossed R38H02-Gal4 to UAS-TrpA1, activated for 12 hours, and compared their performance to genetic controls (**Figure R5,** below). We did not see any effect on attention. This does not contradict our findings, it just suggests again that the R4 neurons are probably not causal on their own for this readout, but still reflect the starvation state of the animal. We are hesitant to pursue this line of circuit analysis for now, because it is not pertinent to our story. We have therefore opted not to incude Figure R5 in our revised manuscript.

**Figure R5. TrpA1 activation of R4 neurons does not alter the visual attention phenotype.**

Flies were reared at 19 degrees C and shifted to 29 degrees C 12 hours prior to experiment. n=8 per genotype. One-way Anova with Tukey correction was used for comparing different conditions. . (R38h02-Gal4/+, black and UAS cTRPA1 RNAi/+, grey; R38H02 Gal4> UAS-dTRPA1, orange. n.s.= not significant. Error bars show SEM.

Minor comments:
1) Were virgin female flies used for all the experiments? It’s specified in some sections of the Methods but not in all.

Yes, all flies tested were virgins and this is now made clear in the Methods (line 513).

2) Lines 132-133 (referring to Figure 1B), the authors write that upd2 mutants show “no significant change in total food consumption,” but from the data points shown it looks like the experiment was underpowered. The authors should not make any inference about the lack of “significant change” in this case.

We have now increased our n for these experiments and still conclude that there is no significant difference.

3) Lines 138-139, the phrase “this suggests that upd2 mutants are feeding when they normally should be achieving most of their deeper sleep” implies that the flies are sleeping less during the period of hyperphagia because they are eating instead of sleeping. However, the sleep loss occurs mostly during the daytime instead of nighttime, and the latter is when the mutants overfeed. The statement should be rephrased or clarified.

Sleep duration during the night is not as severely impacted as it is during the day. However, their sleep is nonetheless fragmented (See Fig. 1L) and lighter (Fig. 3D,E). We have adjusted this sentence to not imply that their feeding is displacing their sleeping (lines 148-149). These are mutually exclusive behaviours anyway.

4) Figure S1 does not “exclude the possibility of a circadian influence (Line 137).” The fact that the feeding pattern (nighttime hyperphagia) persists in constant dark actually suggests that the phenotype has a circadian component, per convention, as I understand it, in the field of chronobiology.

The Reviewer is correct. We have adjusted this sentence accordingly (lines 145-148).

5) Does Figure 4D show a control fly, or a upd2 KD fly? Also, since heat plots are being used to help justify the feeding estimates, heat plots of both control and experimental flies should be shown for qualitative, visual comparison.

Figure 4D shows a control fly. This just exemplifies the tracking system, it is almost impossible to tell # of feeding count differences with a heat map like this (recall, our metric takes account of more than just dwell time near the food), so not really useful to show here. If the Reviewer is curious about what these integrated heat maps can reveal on their own, we would point them out to our new Fig. S7B, where we used this approach to quantify feeding in starved flies (as requested). Here, with feeding pretty much doubled or completely absent, we can begin to see a difference between the conditions by simply looking at the heat map. The Reviewer can imagine that a more subtle difference, as for the data in Figure 4E, would be harder to appreciate. We can add average heatmaps for all of our strains here if asked to do so, but they will all look pretty much the same. It’s the metrics within that matter.

6) It has not been shown that upd2 acts through domeless in the Ilp2 neurons to modulate sleep, feeding, and visual attention phenotypes, as genetic interaction studies are missing. I advise the authors to either tone down the language or perform additional experiments to validate the genetic interaction. An ideal experiment would be to show that expressing upd2 in fat body rescues the behavioral phenotypes in upd2 mutants but not when dome is downregulated in Ilp2 neurons. Alternatively, the authors could show that dome RNAi has no additional effect on the behavioral phenotypes in the upd2 mutant background, although this study would not be as definitive.

As discussed above, we found that expressing *upd2* in the fat body was lethal in adults. This made it hard to proceed with the kind of experiments proposed here by the Reviewer. We feel that consistent phenotypes across the board from *upd2* mutants to *upd2* knockdown to *domeless* knockdown in *ilp2* cells provide an already compelling story to guide future research, and we are careful about not overstating our case (e.g., in the title) about necessity and/or sufficiency regerding the *ilp2* cells for example. We have therefore adopted the advice of the Reviewer to tone down our language (e.g., lines 502-505) throughout.


**Reviewer #2**
In this manuscript, Ertekin et al. uncover and investigate the link between Upd2 and sleep. The work rotates around the hypothesis that upd2 delivers a systemic satiety signal that, when missing, changes the feeding and sleeping behaviour of the flies.

A consistent effect on visual attention is also one of our important findings. We have rarely encountered manipulations that ‘improve’ attention, and the fact that two related upd2 manipulations (*upd2* and *domeless* knockdown) had the same unusual phenotype is striking. Our study is therefore more than about the link between *upd2* and sleep, it is also about how attention and feeding-related behaviour changes when these animals are awake.

The manuscript has several novel findings, with the main one being the characterisation of an unusual sleep phenotype of the Upd2 mutant and the altered feeding pattern, which shows a preference for nighttime feeding rather than during the day. Some of the findings are particularly intriguing: namely, the demonstration of increased activity in the R4 neurons in the brain of fed Upd2 mutant flies, which resembles that of a starved control fly, rather than that of a fed fly and the finding that upon knockdown of domeless (the Upd2 receptor) in Insulin-like peptide-2 neurons in the brain, the sleep and feeding phenotypes also resemble those shown with the Upd2 mutant.

As mentioned above, the attention phenotypes are also recapitulated in both knockdowns.

However, I am afraid the main message of the paper is unfocused and the data are not convincing enough to support the main claim - if anything, they clearly reject it. Overall, the sensation is that there is strong disconnect between the line they authors chose to push and the actual results.

I find there are three main issues:

1) I am certainly not convinced that upd2 delivers a satiety signal. Simply put: a fly that is always hungry should eat more. upd2 mutant flies, instead, eat less and are smaller in size (however this latter is likely to be an not-discussed developmental effect).
Clearly there is a sleep phenotype during the day in udp2 mutants, and there is a different relationship with foraging but none of the results shown is actually compatible with lack of satiety signal - if anything, the opposite is true.

Increased activity of the R4 neurons is compatible with a starvation signal. This aligns with previously published work (Park et al., 2016). However, we concede that before this result, the Reviewer is correct in questioning our terminology, so we have changed our language in the text accordingly and reserved the question of satiety signals for the discussion.

The Reviewer suggests that *upd2* mutants should be eating more. First, flies probably eat as much as they can, so there may be a ceiling effect. What is more interesting here is if they display more feeding-related behaviours. This is exactly what we find, when we move beyond the Café assay and look at feeding-related behaviour in knockdown animals in our open field arena. In *upd2* and *domeless* knockdown animals, the number of feeding events *increases* during the day and the night (see Fig. 4F and Fig. 7E). It is however only significant against both genetic controls for nighttime feeding. The data in Fig. 4F quite convincingly show a generalized increase in feeding-related behaviour, even if one daytime control is not significant. We do not know why the Café assay did not show a similar increase, but again this may be due to a ceiling effect, or may have to do with the food quality (sugar water versus standard fly food), or the finer-grained behavioural analysis available for individual flies in our open field feeding assay (see discussion below regarding this assay). The important conclusion is that the manipulated flies are displaying more feeding behaviour. There is only one experiment where this does not hold, and that is daytime Café for the *upd2* mutants (Figure 1B), but in total darkness food consumption in the mutant trends to more (Fig. S1). Everywhere else, *upd2* manipulations shows equivalent or more feeding behaviour.

2) most of the putative "feeding analysis" in the paper rely on positional tracking. This is clearly the non-optimal tool for this story. Yes, flies seem to spend more time by the food but that does not mean they eat more (in fact, CAFE assay clearly shows they don't). Taken together, the results make me think that the udp2 phenotype is actually *not* a starvation phenotype: perhaps it is linked to food foraging for other reasons? flies use food to regulate their social interaction or to lay eggs. If the authors are interested in finding a mechanistically link, I suggest they look in that direction. If they want to continue on the starvation hypothesis I am afraid they would have to provide more convincing evidence. Perhaps using the flypad from the Ribeiro laboratory in Lisbon may shed some light.

The Reviewer is correct in suspecting that feeding-related behaviours are affected. This is exactly what our new assay is meant to detect: feeding events are not only about dwelling near the food cup, there are a number of other movement criteria that have to be satisfied to count as a feeding event (detailed in the Methods). We have now provided starvation data to further validate our assay, showing a doubling of the number of feeding events in starved flies (Fig. S7B). While it is true that we do not know how much food is actually ingested, we would counter that does not matter because it is the feeding behaviour (visits to the food cup) that is of interest to this study and aligned with our altered attention phenotypes. Indeed, that is the direction our study takes (visual attention behaviour), rather than the metabolism angle. We nevertheless feel confident that our feeding-related metric correctly estimates food consumption. The Reviewer might want to compare the behavioural estimates in Figure 4F with food consumption in Figure 2A (for *upd2* knockdowns), or Figure 7E with Figure S11 (for *domeless* knockdowns). The graphs seem convincingly similar to us.

3) the RNAi phenotype in the fat bodies does not recapitulate nor phenocopies the mutant phenotype, neither in terms of sleep nor in terms of foraging. This adds a level of confusion that leaves the reader disoriented, especially because the authors claim otherwise.

We are puzzled where the Reviewer is confused. Figure 2C (knockdown sleep data) does phenocopy the mutant sleep data (Figure 1G): day-time sleep is severely reduced and night time sleep only lightly. That effect sizes are not as large in the knockdown is probably attributed to penetrance. But the effect is the same. By foraging, the Reviewer is probably referring to the Café results? Here it is true that there is some discrepancy, with less daytime feeding. But total food consumption is similar to controls, also in DD trials (Fig. S1). There is no discrepancy at all between the *upd2* and *domeless* knockdown results, for any phenotype.

4) I don't know what to make of the increase in attention phenotype shown in figure 6. Perhaps is interesting but, again, it is based on too many assumptions. One important assumptions the authors make is that because upd2 flies sleep less than control, they may be sleep deprived (line 421). I would argue the opposite is true: they sleep less because they need less sleep. When we are comparing two different genotypes, the fact that one sleep less than the other says nothing about sleep deprivation state. So in conclusion, ok that udp2 KD have a slightly better attentive performance but how does this relate to sleep at all?

The Reviewer is correct to doubt that the *upd2* mutants are sleep deprived. This is also what we thought, which is exactly why we tested this assumption by providing them with more sleep (via the sleep drug THIP), to see if this ‘corrected’ their hyper focussed attention phenotype. It did not. This is an important result (now Fig. 7). If their attention phenotype was a maladjustment due to insufficient sleep, then providing mutants with more sleep using this method might have corrected it, as shown previously (Kirszenblat et al, 2018). Rather, *upd2* mutants seem to need less sleep, and one consequence is to be hyper focussed. This is in marked contrast to sleep-deprived wild-type flies, which are less focussed (more distractible), which we reported in Kirszenblat et al, 2018. That *upd2* mutants do the opposite while also sleeping less is precisely what makes our study interesting. We have tried to clarify this striking result a bit better in the discussion (lines 577-599).

Additional comments on figures are as follows.

Figure 1
• upd2 mutant flies are smaller (how smaller? Please do not make quantitative claims without quantifying). A comparison should be made between the Upd2 mutant line with control flies which have been matched for size to rule out any effect that may result from a smaller and slimmer fly just showing a different feeding pattern or sleep pattern.

• An additional control of wild-type flies which have been starved could serve as point of reference for the claims.

Physical difference in the mutants have been described previously (Rajan & Perrimon, 2012) and we see the same. We did not feel the need to replicate these measurements, as we trust these are the same mutants.

Figure 2
• The controls used, particularly those in Figure 2A, B and C seem to show significant differences between each other - please include full statistics.

We have indicated the significant effects, if there were any. The full statistics can be found in our deposited files. The specific statistics refered to are in the metadata provided for Figure 2.

Figure 3
• I am personally uneasy about dissecting sleep parameters such as sleep bout lengths because we have no idea whatsoever of what they mean - if anything. I understand the field for some reasons like those, so it's fine to have these measures. However, I do not think one can talk about "sleep quality" as a substitute for longer bouts. This is a tautology that we should not promote in the field. The authors do this multiple times in the manuscript. In fact, their argument is that udp2 flies have worst sleep quality but at the same time they show they perform better in cognitive tests.

The Reviewer may not have understood our sleep intensity measures in Figure 3, which follow from multiple publications (that we reference) where these are more thoroughly explained (e.g., Faville et al, 2015). Briefly, these are not simply sleep duration bouts. We measure sleep intensity by probing for behavioural responsiveness to mechanical stimuli, and this can happen any time between 0 and 60min. How responsive they are (how deeply asleep they are) depends to some extent on how long they have been asleep, especially at night (see Fig. 3E). The binned data in Fig. 3B is just to show that flies were probed for all of the different sleep durations. There are comparatively fewer probing events for 30-60min during the day because fewer flies ever sleep that long during the day, on average. Our measures stop at 60min because our probing events are hourly; how long flies have been asleep before the hourly probe is stochastic. We now provide some additional Methods (lines 667-672).

We hope we have clarified better our sleep intensity assay, so that the reader can appreciate why allude to sleep quality. There is no question that *upd2* mutants and knockdown animals have more fragmented sleep (Fig. 1J-L) and are easier to wake up (Fig. 3D,E). Following Reviewer #4’s questions (below), we now provide the sleep consolidation / fragmentation data for the *upd2* and *domeless* knockdown strains as well (Figures S5 and S10). It all quite consistently shows a difference in sleep quality in these animals. That this should be matched by ‘improved’ attention is again precisely what makes this study interesting. We are however cautious in not interpreting this as ‘better’ performance, and provide quite a bit of discussion on what this ‘improved’ attention phenotype might mean ethologically (lines 577-599).

• In figure 1K-L, the authors show that udp2 mutant flies have shorter bouts. If, like the mutant line, the knockdown line shows bout lengths similar to that of the mutant, measuring arousal past 31 minutes during the day would lead to measurements being taken from a non representative sample of bouts, as no individuals from the mutant line had bout lengths longer than 20 minutes (similarly with measurements in the night time). Incidentally, the sample size used for these experiments is also not stated in the manuscript.
This figure would benefit firstly from the same analysis done with the mutant line where measures of bout duration and number of sleep bouts are taken first before measuring arousal.

Sleep in *upd2* mutants was more severely affected compared to the knockdown strain. This is not surprising as the knockdown experiments were performed to circumvent developmental effects (Yolk-Gal4 expression is only in adults). As can be seen in Fig. 3B, knockdown flies are sleeping past 31 minutes, even during the day. There are fewer instances though, as expected, but enough to say something about sleep intensity differences for most time bins.

The dataset in Fig. 3 are from the same flies as in Fig. 2, which we have noted in figure legend. We have now added the sample sizes under this figure as well.

Figure 4
• The assay used here for feeding is not state-of-the-art and is confounded by too many issues. See major point #2

We have addressed this concern in our reply to major point #2, above.

Figure 5
• This is interesting. Figure 5D would benefit from adding a starved version of the Upd2 mutant in addition to the fed version. Also, quantitative palettes should be used so that the highest level is never reached and GFP intensity appears to be almost saturated in this image - it would be interesting to see if the starved Upd2 mutant could give an even greater intensity.

We thank the reviewer for the interesting suggestion. We have performed the experiment and saw no difference between fed and starved *upd2* mutants. These new data have been added to Figure 5. The colour palette that was used reflects the range we saw, from fed wild-type flies to *upd2* mutants. We aren’t sure that adding numbers to panel D will be informative.

Supplementary Figure 6
• This figure would benefit from quantification of food intake with the drug. If the drug also changes feeding behaviour or increases how much food the flies eat, they may show greater sleep from a postprandial response.
• If using Gaboxadol changes the amount of sleep but does not change attention, this could be further support for the phenotype being one of increased foraging rather than a starvation phenotype.

Several labs now have gone through considerable effort optimizing the use of THIP to induce sleep in flies (e.g., Dissell et al, 2015; Berry et al, 2015), to for example account for potential differences in feeding while on the drug. The conclusion is that feeding is normal.

We agree that increased foraging is a likely consequence of our genetic manipulations, consistent with the attention phenotype we have uncovered, and with the increased feeding-related behaviours we have observed. Quantifying foraging is an interesting future direction.

**Reviewer #3**
This is a very creative and innovative manuscript that describes a relationship between sleep and attention. A number of conceptual aspects are highly novel and will broadly influence the field including the identification of upd2 as a molecular link between peripheral energy stores and central brain regulation of sleep, and the finding that increased arousal in upd2 mutants may promote attention, despite a reduction in total sleep. The manuscript is well written and technically sound. I do believe there are a number of addition experiments that are needed to support or clarify the conclusions in particular see points 2,3, 4, 6, 7.

1. While there is evidence that upd2 functions as a homolog of leptin (Rajan et al, 2012, Rajan et al, 2017), my understanding is this is still relatively controversial and it may be safer to describe upd2 as a secreted cytokine, then discuss its leptin-like role in the discussion section.

We appreciate the advice and have altered our language throughout, starting with the title.

2. The feeding data in 1B are non-significant, but the absolute values are different by nearly 50%, suggesting the need for increased statistical power. Also, the legend states N=40, but there are only 5 data points. Please clarify.

We have performed additional experiments to provide a more appropriate level of statistical power as suggested. The result is still non-significant. We have also adjusted the legend.

3. Line 183. ‘any effects on sleep were inconsistent compared to controls.’ It is unclear what this means. The best control is the RNAi line alone, which provides a phenotype, raising the possibility that upd2 functions in the brain. It may be worth testing with additional GAL4 or fat body drivers to clarify these results.

We have adjusted our language to reflect the possibility that upd2 could still be expressed in neural tissue, in addition to the fat body, to affect sleep (lines 237-239. However, the absence of associated feeding or attention phenotypes in the pan-neuronal knockdown (Fig. S4 & S9) suggests that the source that is of greater interest to our study remains the fat body.

4. The authors bin arousal threshold in 1-30 or 30-60 minute bins, but I believe their earlier work has shown that the switch from light to dark sleep occurs early. The conclusion that sleep intensity is reduced in upd2 mutant flies would be improved by greater temporal resolution.

We have increased the temporal resolution of these experiments to 10min. The conclusions are the same, but we agree that this shows better the value of sleep intensity data.

5. Based on the text, I do not understand the contributions of Figure 4. First, other systems have been devised to simultaneously measure sleep and feeding e.g. ARC assay. Second, the system cannot distinguish whether the animal is eating, only whether it is near the food which can be detected using the DART. If the purpose is to confirm results in a different arena, then this is valuable and should be stated. Otherwise, please clarify how this improves previous figures.

All reviewers questioned the validity of this combined sleep/feeding assay, and we have tried to satisfy these concerns in our replies, above. Briefly, it is correct that we measure feeding behaviour rather than food ingestion *per se*, but we feel that this is appropriate for the purposes of this study. Feeding behaviour (# of feeding events) is more than just dwell-time near the food well, it involves a couple dynamic metrics that are described in the Methods, and available in the newest version of DART. Our new paradigm allows us to effectively link sleep and feeding behaviour at the level of individual flies, which is crucial for coming up with the CaLexA kind of data shown in Figure 5. Hence, it was crucial that we first convincingly show that this new assay recapitulates the sleep phenotype (Fig. 4A,B) and the feeding phenotype (Fig. 4E,F). We feel that this simple way to estimate feeding behaviour in combination with sleep/locomotion data will be beneficial for the field.

6. In figure 5 it would be useful to show CaLexA in the fed and starved state for both the mutant and wild-type. In addition, this would be useful for the R4 correlations in 5B.

We have done the additional experiments on starved *upd2* flies as suggested. They show no difference in R4 neuron activity than fed *upd2* flies. It should be noted that the results in Fig. 5E are from a different dataset than 5B (i.e., a different antibody cohort). The flies in 5E were not tracked for sleep or feeding behaviour. The goal there was to control for staining effects by preparing and imaging these together. We try to make this clearer in the Figure legend.

7. Why was the attention assay only performed in RNAi knockdown flies. It is incongruous to have the R4 experiment exclusively in mutants and the attention work in knockdown experiments. The easiest way to rectify would be to perform the attention experiments in mutants. I also do not understand S6. The sleep measurements with THIP are in mutants, but the attention assay is in the RNAi knockdown.

Unfortunately, the eye colour background of the *upd2* mutants precludes doing any visual attention experiments. Hence, all of our RNAi knockdown experiments are in an outcrossed w+ background. This is why the rest of the paper focusses on this genetic strategy, because visual attention is central to our story.

We have now repeated the THIP experiments on the knockdown strain and genetic controls, and found that sleep was increased for these as well (see reply to a similar request from Reviewer #1, above). We feel this is an important result that follows more logically from Figure 6, so we have now included it as a main figure (new **Fig. 7**).

Minor comments
1. Line 54: ‘the phrase ‘maintain well-tune cognitive processes’ is vague.

2. The first paragraph lacks citations. Even though the concepts are generally well-accepted it would be helpful to point readers to relevant literature.

3. It’s not clear why the day and night are labeled by yellow and blue. Especially since others have used this to denote fed-starved. May be better to use white and black boxes to maintain consistency with the circadian literature.

Adjusted and corrected 1-3.

4. Please clarify line 183. If they are awake/foraging, why not feed?

Perhaps you are referring to line 157 in our original submission? “However, rather than feeding or sleeping during the day, the mutant flies are mostly walking persistently back and forth in their chambers with constant speed (Figure S2)”.

It’s a good question, about feeding. The knockdown strains are perhaps more informative in this regard, as we show data for food consumption, feeding behaviour, sleep, and attention. Food consumption (via Café) is higher than control flies at night (Fig. 2A), and this is matched by more feeding events at night (Fig. 4F). In both cases, daytime feeding is also higher, but not significant. So they are actually feeding more. How the ‘improved attention’ phenotype manifests in nature is of course an open question, but we suspect that this represents increased attention to stimuli that are most salient for flies (the 7Hz flickering bar we use in the attention experiments is, incidentally, highly salient – see Kirszenblat et al, 2019), to ensure that they focus and find food, rather than be distracted by other less salient stimuli. We have since done some virtual reality arena experiments with these knockdown strains, and it looks like they are less novelty-seeking than controls. This raises the intriguing possibility that starvation signals decrease cognitive flexibility. We will be describing those results and hypotheses in a subsequent publication more centred on these cognitive questions.

5. In methods, please reference the w+ line used for outcrossing.

Done.

**Reviewer #4**
In this manuscript, the authors present an interesting set of experiments that seek to understand the relationships among sleep, physiology, and cognitive function. While we find the questions highly relevant and the data intriguing, there are several conceptual concerns that limit our enthusiasm for the manuscript in its present form. Some involve what we believe to be over interpretation of the data, which, in our opinion, obfuscate the main thesis of the paper and logical thread of the work as well as confuses direct observation with interpretation, thereby potentially limiting its overall impact. Other concerns center on what might be considered insufficient depth of mechanistic understanding of key components of their model. Significant textual revisions are recommended, as are a small number of additional experiments.

The title of this manuscript implies discovery of a link between the expression of a satiety signal (i.e., upd2) and sleep need and visual attention. These reviewers agree that this is a well-supported and interesting finding. However, in our opinion, this message is diluted throughout the manuscript by the authors persistent presentation of conjecture as established fact (e.g., by equating upd loss with starvation), by conflating key ideas (e.g., sleep need is not the same as sleep loss), and by over-interpretation of behavioral data (e.g., positional preference does not imply feeding). These positions are unnecessary in the Introduction and Results, and they actively hinder the reader’s ability to carefully interpret the experiments. Such conjecture of the data should be confined to the Discussion.

Assertion of a starvation-like state: The authors repeatedly state that loss of upd signaling emanating from the fat body is responsible for a “starved-like” state; a state the authors believe mimics the starvation-induced sleep deprivation phenotype widely reported by others. Maybe, maybe not. True, some phenotypes of upd loss are similar to those in starved animals (e.g., decreased sleep duration and quality). But others are not (e.g., upd LOF flies don’t eat more, starved flies do). The timing of when upd mutants feed is altered, but this may indicate changes in sleep-wake behavior and/or in baseline metabolic state. We agree that it is intriguing to consider that these animals are “genetically starved,” but we are not convinced this is the case. We propose two possible ways to address this. First, the authors could simply move the discussion of a possible starved-like state and its implications to the Discussion and maintain a focus on describing the results precisely
(e.g., that upd loss is causing the phenotypes, not starvation). Whether the flies are starved does not impact the fact the sleep and attention are affected by upd. Alternatively, the authors might provide more convincing evidence of perceived starvation such as:

(1) Determining whether starving upd mutants or yolk-GAL4 > upd-RNAi flies alters sleep, feeding, and attention phenotypes: if loss of upd achieves a starved-like state, then further starvation would not significantly alter these phenotypes

The other Reviewers have also questioned why *upd2* mutants don't eat more, if they are indeed ‘genetically starved’. Actually, all of our data suggests that downregulating *upd2* and *domeless* leads to increased feeding as well as increased feeding-related behaviours (Fig. 2A, Fig. 4E&F, Fig. 7E, Fig. S4, Fig. S12). Only the *upd2* mutant on an LD cycle shows less daytime feeding, balanced by more night feeding to equal the total consumption seen in controls. In DD, the mutant actually trends to eating more than controls (Fig. S1). Our Café data for *upd2* knockdown in the fat body also shows greater food intake at night, and a trend for greater food intake during the day (Fig. 2A). We now have done the same Café experiment for *domeless* knockdown in the ilp2 neurons and see increased feeding in these flies as well (Fig. S11). When we track feeding behaviour (our open arena assay), all of our data point in the same direction: *upd2* knockdown flies display significantly more feeding events at night and a trend to more events during the day (Fig. 4E,F). As for the Café results, the data for feeding events for *domeless* knockdown is almost identical (Fig. 8E), with increased feeding events at night and a trend to more feeding during the day. Again, the only experiment that did not show generally increased feeding was Café data for the *upd2* mutant, which unfortunately may have set the wrong tone for the paper (Fig. 1B,C). There are many reasons why the knockdown experiments might be more believable, starting with our understanding that Yolk-Gal4 expresses only in adults (Georgel et al., 2001). Therefore, we believe that the totality of our data supports the observation that knockdown of this cytokine signal from the fat body, from upd2 secreted in the fat body to its *domeless* receptor in ilp2 cells to increased R4 activity leads to a starved-like state. Nevertheless, we appreciate that referring to a ‘satiety signal’ or a ‘starvation cue’ might be premature in the early parts of the paper, so we have modified our text accordingly throughout, including the title.

Regarding the Reviewer’s question about the effect of starvation in u*pd2* mutants, we have done that experiment as suggested. Starvation does not further decrease sleep duration in *upd2* mutants. We have added these new data to Fig. S3, and now also refer to this evidence in the text (lines 191-192).

(2) Asking whether conditional loss of upd function using tub-GAL80ts or tub-GeneSwitch affects baseline sleep, feeding, and attention measurements relative to baseline prior to the manipulation. Furthermore, this strategy would also help clarify whether the phenotypes reported in the paper are caused by the current state upd signaling or instead cause by loss of upd signaling throughout development.

Our usage of Yolk-Gal4 already addresses this concern, it only expresses in adults (Barrio et al., 2014; Georgel et al., 2001). We have verified this ourselves by expressing GFP in Yolk-Gal4. GFP expression is only evident by around day 3 of adulthood. We can provide these images if requested, but prefer referring to published work in this regard, as this is not novel.

Position preference as an “accurate estimate” of feeding: Similar to the claim of genetic-starvation, the authors use positional preference to infer “feeding events.” These two may indeed be correlated, although is many instances they are not. Either way, the authors should simply state these data as “positional preference” or “time spent near the food” in the results to avoid the indication that this assay explicitly measures feeding. The lack of flies coming to the food cup when it is covered with parafilm does not provide evidence for or against what they do when they are there, and the observation that they spend similar amounts of time there when it is filled with agar seems to us to argue against the notion that this is an accurate measure of feeding. Either way, its accuracy has not been established.

Please see our answer to similar concerns from the other Reviewers, above. In addition to clarifying this approach (i.e., it isn’t only dwell-time near the food), we have also conducted additional experiment to verify its validity (e.g., testing hungry flies, Fig. S7B). Following the Reviewer’s advice, we have also modified our language where appropriate to refer to feeding-related behaviour rather than actual feeding events. We do not feel that this compromises our story, because the interest here is indeed in food-seeking behaviour, not how much flies have actually ingested.

Lack of connection between DILP2 and R4 Neuron Activity: Given the observations presented in Figures 5, 7, and associated supplemental figures, DILP2 neurons must somehow communicate with R4 neurons to regulate their excitability and, in turn, form the basis of nutritional state control over sleep behavior. The manuscript would be substantially strengthened if the authors provided data illuminating the relationship between these neurons, which can be done either by (1) neuroanatomical characterization of possible synaptic connections made between DILP2 and R4 neurons or (2) determining whether R4 neurons respond to artificial activation/inhibition of DILP2 neuron activity using the CaLexA approach.

We agree that these are interesting future experiments. How exactly the ilp2 neurons communicate to the central complex is of particular interest, and the subject of another study we are currently putting working on. We respectfully disagree that this complex circuit analysis belongs in the current study; the R4 neurons in the current study are only being used as a ‘sanity check’ for our view that these flies are ‘genetically starved’, not as an invitation to explain the functional link between ilp2 neurons and R4 neurons. That is a massive project, which cannot realistically begin at Figure 9 in our current study.

Phenotype in mated female and/or male flies: It is worth noting that only virgin female flies were tested throughout the manuscript. However, it is widely understood that sleep/feeding behavior is not only sexually dimorphic, but is also strongly modulated by the reproductive status of female flies. The authors should strongly consider investigating the upd mutant phenotypes in mated females and male flies to better establish the relevance and generality of the observations.

We agree that establishing the generality of this effect across sex and reproductive status is valuable. However, especially for a sleep study, it is not recommended to use non-virgin females, as this includes a heterogeneous population that may confound the results. Additionally, egg laying behaviour and even the eventual larvae may confound the results. Hence, we have stuck to virgin females to provide a convincing dataset and would certainly be interested in exploring if our results hold for male flies as well. However, we feel that that is a different kind of study with different questions.

Requirement of amino acid for feeding/sleep phenotype: This reviewer noticed that most of the feeding measurement were performed using 5% sucrose food. However, sucrose-only food does not support normal lifespan or physiology and there is increasing evidence supporting the importance of specific amino acids influencing feeding-dependent effects on sleep-wake regulation and homeostasis. While it is, perhaps, extreme to ask that these experiments be repeated under standard nutritional conditions, this limitation should be clearly stated and discussed in the Discussion section.

Only the Café experiments were performed with liquid (5% sucrose) food. For all of our other experiments (i.e., the open field arena involving a food well in the middle), we used the same standard (solid) fly food that the flies were raised on, described in the Methods. That we saw very similar results for feeding in the Café assay and the open field arena for *upd2* and *domeless* knockdown suggests that the latter is providing a fairly accurate estimate of feeding.

Differences between mutant and tissue-specific RNAi knockdown observation: The authors generally conclude that upd signaling from the fat body is responsible for its effects on feeding and sleep (lines 200-201; Fig 2). However, liquid feeding amount (e.g., daytime feeding) and sleep measurements (e.g., modest effects during nighttime; not significant across all controls) appear to be different between the mutant and fat-body knockdown strategies (Fig 1C vs. Fig 2A). The authors should comment on this discrepancy. As a side note, data showing that the upd2-RNAi is effective should be presented, either by determining whether ubiquitous knockdown of upd2 phenocopies the mutant phenotypes and/or by measuring the extent of upd transcript reduction caused by fat-body specific RNAi knockdown.

Please see our reply to a similar concern from Reviewer 1-major comment 2, above, as well as to this Reviewer’s earlier comments. We concede that the mutant seems the odd one out here with regard to the Café results. However, these are still well-fed flies. We have provided three different knockdown contexts for *upd2*, fat body, muscle, and neurons. We feel that this is a sufficient survey of possible tissues, as a follow up for the *upd2* mutant itself. The fact that our *domeless* knockdown results closely phenocopy *upd2* knockdown in the fat body is also sufficient, we feel, for providing confidence that these results are real.

Sleep fragmentation measurements using tissue-specific RNAi approach: The authors should quantify and present sleep fragmentation parameters, similar to what is presented for the upd mutant in Fig 1H-L, for the tissue-specific knockdown data collected in Fig 2, 4A-C, and 7C-D. The expectation is that night-time sleep is much more fragmented when upd is knocked-down in fat body cells (i.e., increased sleep bout number and decreased average bout duration).

We now provide all the additional sleep fragmentation data as requested by the reviewer, in **Figures S5,S6,** and **S10**. Recall that in Fig. 2 we observed daytime fragmentation, however we were unable to observe night-time fragmentation. Differences in sleep quality at night became more evident in our sleep intensity results (Figure 3).

Additional Comments:
The Fig 1 legend states that “(B) Food intake was measured with Café assay, with 5 flies/chamber over 20 hours (n=40 per genotype)”. However, the number of data points do not match this description (only 5 data points appear to be plotted). The only possible explanation is if each data point represents a unique experiment. If that is indeed the case, that should be explicitly stated.

We have corrected the legend.

In the food intake figure presented in Figure S1, the term “ZT” along the x-axis should be changed to “CT” since the experiment is being performed under constant darkness conditions.

We have corrected this.

Both the interpretation and presentation of Figure 7 should appear before Figure 5 for the sake of continuity. According to the authors’ proposed model, upd signaling from the fat body is received by DILP2 neurons, which in turn somehow sends direct/indirect nutrient status information to R4 ring neurons to regulate sleep. The way it is currently presented in the manuscript, it seems like the R4 ring neurons come before the DILP2 neurons.

Please see our reply to the question about ilp2-R4 neuron circuit analysis, above. We would prefer to keep the R4 results where they are, because their intention is not as a starting point for circuit analysis, but rather as a check for the upd2 mutants being ‘genetically starved’.

Additional detail should be provided in the Methods section regarding measures of sleep intensity, specifically the calculation/algorithm that was used to determine whether a fly responded to a stimulus.

We have provided some more detail here as requested (lines 667-672), but also point the reader to our publications where these methods are elaborated. We appreciate that this is a relatively novel approach to studying sleep, and that not everyone is aware of it.
