# Supplementary material for: Down-regulation of a cytokine secreted from peripheral fat bodies improves visual attention while reducing sleep in Drosophila
Source: PLoS Biol. 2020 Aug 3;18(8):e3000548. doi: 10.1371/journal.pbio.3000548 (PMC7426065; doi:10.1371/journal.pbio.3000548)
Supplement: Attachment — Submitted filename: Reply 2.docx [file pbio.3000548.s015.docx]

Please note: all of the line numbers refer to the Ertekin_R2_legends embedded_clean document.

Rev. 1:
The revised manuscript is greatly improved. Removing the opto experiments and the changes to the text make the paper much more readable. The authors have addressed the critical concerns adequately. I have only two minor suggestions:

1) Page 17, line 459: I don't think this comparison should be made across experiments. If you really want to make this claim, I think the lines need to be tested at the same time, both with and without THIP.

We have changed the wording to say “THIP exposure” rather than “additional sleep” (line 475), as the Reviewer is correct in saying that we did not technically measure sleep and attention in the same individuals. THIP exposure by feeding reliably increases sleep in flies, and the exposure time was identical, so we do not think that this is a problem. Rather, testing visual attention in flies that have been confined to Trikinetics tubes is problematic (as explained in Kirszenblat et al (2019), *Sleep*, which is why we had to use separate groups of flies.

2) Page 18, line 484-5: The subheading overstates the findings. I think the subheading should be revised to be more conservative, similar to the figure legend title.

We did exactly as suggested by the Reviewer, and changed the subheading to be more conservative (line 501).

Rev. 2:

[Point 1] True you show that one circuit namely R2 neurons which respond to starvation have increased activity. There are many many more circuits involved. I refer you to Krishna Melnattur, Paul Shaw 2019 review on the subject. What about LHLK neurons? PAMs? IPCs/DILPs, NPF? I would expect a much more comprehensive testing of your hypothesis. As it stands it is a big (yet in my opinion unnecessary) part of the paper which is not adequately addressed. (see general comments at the end for clarification)

While we agree that there are many other potentially interesting circuits to look at, a comprehensive survey of feeding-responsive neurons was never a goal of this study. We focussed on the R4 neurons for two main reasons. First, because they had been previously identified as starvation-responsive using CaLexA (Ref. #64), and therefore served as a ‘sanity check’ for our hypothesis. Second, we were interested in the ellipsoid body (EB) because of its demonstrated role in visual behaviours and sleep (Ref. #63). We try to make that original rationale a clearer now. To help convince the reviewer that the R4 CaLexA results are relevant, we show below CaLexA data for other EB circuits in starved versus fed animals (Figure R2.1). Only R4 neurons show increased activity with starvation. That these are the exact same neurons that have been highlighted by others as starvation-responsive seems convincing to us that, in an *upd2* mutant background, they indicate a chronically starved state. We have however toned down our ‘genetically-starved’ language throughout this second revision, following closely the advice of the Editor. We also now refer to Melnattur & Shaw (Ref. #62) as suggested, to highlight that other circuits might be considered (lines 359-361).

**Figure R2.1**: CaLexA imaging for 4 different ellipsoid body (EB) circuits, in starved vs fed flies. Data in R38H02-Gal4 are taken from Fig. 5 in the manuscript. The other EB experiments, shown for comparison, are not in the manuscript. Only the R4 circuit showed significantly increase calcium activity in starved animals. Error bars are SEM.

[Point 2] I have a problem with the feeding aspect of the paper in general. Please see other comments that address this concern

We now provide additional analyses (Supplementary Figure 7C-E, shown also below in Figure R2.2) where we have gone back to the original movies and *manually* verified that feeding events have been correctly detected, and that visual verification is consistent with the automated tracking of feeding events. Flies simply walking past the (solid) food cup are not eating, so this is not counted as a feeding event. Flies stopping at the food cup for >30s are eating. Flies totally immobilized at the food cup for extended periods are not eating, they are sleeping, so not counted as feeding. Our automated tracking of feeding-related behaviour accurately describes feeding-related behaviour. We feel most readers will accept this, especially now with the added manual verification data. We elaborate on these additional confirmatory experiments in the text (lines 328-338).

**Figure R2.2**: (C) Representative images from the video recordings. The bottom image is showing a fly feeding. (D) Visual annotation of the number of food visits displayed a significant increase in starved flies. (E) The average duration of food visits per fly was not significantly different between control and starved flies. Data are individuals.

[Point 3] There is a problem with replicating feeding results in this paper. In figure 1a Upd2 mutants have nighttime hyperphagia but no overall effect over 24hrs. In Figure 2a there is an overall difference between one control and KD but not the other in total intake. In figure 4E KD is different from one control group but not the other and in 4F KD are hyperphagic at both day and night. I am struggling to draw a conclusion here. Also from what I can tell/ it is not clear if you are using different diets in CAFÉ assay/open field arena and in DART assay. Are these comparable- please clarify?

The diets used in the different feeding assays are mentioned in the text and described in detail in the methods. Only the Café experiments used a liquid food source; all other experiments (e.g., circular arenas) utilized the same solid food source, which is the standard fly food described in the methods. It is therefore convincing to us that hyperphagia results were replicated using either type of assay: significantly increased night-time feeding was found by both Café and circular arenas for both upd2 and dome knockdown animals (Fig. 2A, Fig. S4A; Fig. 4E&F). It is true that the upd2 mutant is different for daytime feeding, but it also shows increased night-time feeding, just like the knockdown animals (Fig. 1C), and the daytime effects may be a daylight related effect that is peculiar to these mutants, as it goes away under DD (Supplementary Fig. 1). It is also true that there is more variability with daytime feeding across all strains, with some non-significant effects against one control. But we never make a point about this being significant. Rather, we focus on night-time hyperphagia as being a common feeding phenotype in the three strains (upd2 mutants, upd2 knockdown in the fat body, and dome knockdown in the Ilp2 cells). This is shown by both Café and feeding-related behaviour in the three strains. We remain puzzled why the Reviewer does not see these results as consistent. That there are non-significant effects against controls (e.g. during daytime feeding) says just that: daytime feeding is probably less affected than night-time feeding.


[Point 4] Ok- I understand that people like to use this drug [THIP] but I doubt it recapitulates natural sleep. It may be more akin to anaesthesia. I don’t think this experiment adequately addresses the issue of sleep deprivation but accept this is a tool used in the field.

Our lab studies general anaesthesia, and we can confirm that THIP does NOT act like a general anaesthetic, ever. Rather, it is a drug that has now been shown by several labs to promote sleep in flies, probably something more akin to deep sleep. We are glad that it is gaining acceptance by the field, to study sleep functions in flies.

Additional comments on figures:

[Figure 3] Line 243: Authors say that flies can compensate for sleep loss by exhibiting deeper sleep during shorter sleep bouts yet use a vibration stimulus to probe “sleep depth”. Sleep depth does not necessarily equate to increased quality. For example in mammals sleep is characterised by different stages (REM, non REM 1,2,3,4) which are necessary for different aspects of cognitive performance. REM sleep is though to be important for dissociation between memory and emotional salience for instance, but it is much easier to wake someone in REM sleep compared to non rem stages. Please provide some discussion/caveats to your conclusions here.

We have provided discussion/caveats about this interesting point (lines 593-598), in the discussion (paragraph starting with ‘It is however unclear if upd2 mutants *need* more sleep.’ Incidentally, it is not quite accurate that it is easier to wake somebody from REM, compared to non-REM sleep stages. See for example Dement and Kleitman (1957), or Peever and Fuller (2016); but that is beside the point of our revised manuscript.

General comments from the reviewer:
In general there are some interesting aspects to this paper but the feeding/starvation aspect of it is confusing and weak. For me a simpler and stronger story could be compiled by looking at the effect of upd2 and domeless mutants/KD on sleep, depth and fragmentation. The apparent lack of compensation for sleep loss yet maintenance/improvement in attention in domeless and upd2 mutants/KD animals is the most interesting part. You can discuss feeding dysregulation/starvation in discussion but I don’t think you have adequately demonstrated that you are mimicking a starved state or feeding dysregulation in these animals. As I said previously you are not using state of the art means to measure intake (such as ARC or flypad). In general the feeding phenotypes are weak, upd2 mutants are different sizes and as far as I can tell you do not normalise food intake by body size. I would encourage a rework of manuscript composed of figure 1F-L, Figure 2 C,D,G,H,K,L. Figure 3, Figure 5 (including comparison between control and Upd2 fed conditions but not starved.), Figure 6 and Figure 8. It still works well without feeding side of it.

We respectfully disagree with the Reviewer’s suggestion of removing all of our feeding results from the paper (Café as well as circular arenas). We view this as an unusual request, as the feeding data are what they are and should be available for all to see. We will however follow the advice of the Editor on this point. Following their latest advice, we have now tempered our language about ‘genetic starvation’ throughout the paper. We have also focused on ‘feeding related behaviour’ throughout rather than feeding per se; we have shown consistent results between the different assays (see above); and we have now provided visual validation that the automated tracking of feeding-related behaviour is accurate. We are grateful that the Reviewer feels that our paper works better now, but we fail to see how removing all of the feeding results would make that an even better paper. If some readers conclude that other state-of-the-art measures of food intake in flies would be a good departure point to delve deeper into our findings, they can do that after having appreciated our accessible and novel approach. We suspect the Reviewer may have been more concerned with our previous language (‘genetically starved’), which we now have adjusted, than really asking us to remove all of the valuable feeding data. For a metabolic mutant, these data are extremely relevant.

Other comments
Images in Figure 5D are oversaturated. While the effect is clear from the representative images it makes me nervous about the validity of the imaging technique as a whole.

These are pseudo-colour images. We have not manipulated the raw images to enhance any effects. Imaging criteria were kept the same for all samples, and any comparisons to controls were done under the exact same imaging conditions. This is stated in the methods (line 767).

Where are stats/Ns in 3B? Are all bouts observed in longer bins contributed by one fly? Or many? Ns/bin not reported on graph/ legend, only the number of flies in the experiment in figure legend. “(B) The number of animals in each sleep duration bin for both day and night were similar in both genetic controls (black and grey) and in knock-down flies (orange).” How can this be if in figure 1 you show strong differences in bout number and length? Please clarify. Incidentally if the genotypes are not receiving the same number of stimuli (stimulus/fly/genotype), it is unlikely that they are receiving the “same” stimulus as you may have a problem with habituation. You will need to acknowledge this if this is the case.

We realize this graph might have been confusing, and we have tried to clarify its purpose, in the text (e.g., lines 251-255). First, the results in Figure 3 are for knockdown animals, so more directly comparable to sleep data in Fig. 2, not Fig. 1 (which are the mutants). The graphs themselves represent not the number of stimuli (all flies receive the exact same number of stimuli), but rather the number of flies that were asleep for x minutes (the minutes asleep bin) when a stimulus occurred. Naturally, there are fewer such example during the day, because few knockdown flies ever sleep that long during the day. The main purpose of the graph in Fig. 3B is to show that sleep intensity data in Fig. 3C&D are valid, because they are substantiated by adequate numbers in Fig. 3B. This is why there are no stats in 3B, it’s basically just an N. All of this is thoroughly explained in Faville et al, 2015.

Line 325: why was 30s chosen as criteria for feeding? What is average feeding bout length? Are you not missing shorter feeding bouts? This is why a method like flypad would be far superior in this study.

We agree that Flypad is a sophisticated method for feeding behaviour analysis. However, while it would be very interesting to look at Flypad metrics in the future, it was not the focus of this study. Further our open-field analysis was based on another study by Ribeiro lab (where Flypad was developed). In this recent study (<https://doi.org/10.7554/eLife.19920.001>), the authors combined their Flypad analysis with an automated video tracking and saw that 70-92.2% of the micromovements on food patches corresponded to a feeding bout. Based upon these observations, they conclude that video-based micromovement tracking appropriate to capture the feeding periods the fly spends feeding on a food patch. Our 30seconds criteria was based on their results, as a typical visit for wild-type flies was ~30 seconds. We have since confirmed this with visual observations from our own data (Supplementary Fig. 7C-E).

Rev. 3: Alex Keene
The revised version of this manuscript has addressed all my initial concerns through the inclusion of additional data and changes within text. I have two minor suggestions below that could be addressed at the authors discretion.

1. I prefer the sentence on line 83 to be deleted or modified. Behavioral performance is used in a non-specific way, and one could envision many behaviors (such as foraging) that are better when the animal is not well-fed.

This line has been deleted, as suggested by the Reviewer.

2. Clarity could be added to paragraph on insulin signaling (line 616). Which neurons were found to be Ilp2 targets in larvae? Does the suggestion they communicate 'indirectly' mean non-synaptically or through intermediary neurons? It is also worth noting that there are many other sources of insulin, other than Ilp2 neurons.

We have clarified this paragraph as requested, but also tried to keep it brief as it is the last paragraph of the discussion and we did not want to digress into a larger discussion on insulin signalling at this point.
