# Supplementary material for: Down-regulation of a cytokine secreted from peripheral fat bodies improves visual attention while reducing sleep in Drosophila
Source: PLoS Biol. 2020 Aug 3;18(8):e3000548. doi: 10.1371/journal.pbio.3000548 (PMC7426065; doi:10.1371/journal.pbio.3000548)
Supplement: Attachment — Submitted filename: Response.docx [file pbio.3000548.s016.docx]

Jessica Hyde

Production Team

*PLoS Biology*

1 July 2020

Dear Ms Hyde

We have uploaded PACE-formatted tifs of all the main and supplemental figures, as well as final version of the manuscript that complies with the latest style requests.

We have now also removed the sentence ending with ‘data not shown’ as requested, and accomplished the latest requests from email from 30 June, 2020.

Sincerely yours,

**Bruno van Swinderen**
